# Applications of Grounded Theory Methodology to Investigate Hearing Loss: A Methodological Qualitative Systematic Review With Developed Guidelines

**DOI:** 10.1097/AUD.0000000000001459

**Published:** 2024-04-14

**Authors:** Yasmin Ali, Nicola Wright, David Charnock, Helen Henshaw, Haley Morris, Derek J Hoare

**Affiliations:** 1 National Institute for Health and Care Research (NIHR) Nottingham Biomedical Research Centre, Nottingham, United Kingdom; 2 Hearing Sciences, Mental Health and Clinical Neurosciences, School of Medicine, University of Nottingham, Nottingham, United Kingdom; 3 School of Health Sciences, University of Nottingham, Nottingham, United Kingdom; 4 Nottingham University Hospitals National Health Service (NHS) Trust, Nottingham, United Kingdom.

**Keywords:** Grounded theory, Hearing loss, Methodology, Qualitative research, Systematic review

## Abstract

**Objectives:**

Qualitative methodologies are commonly adopted in hearing loss research. Grounded theory methodology is increasingly used to establish novel theories explaining experiences related to hearing loss. Establishing and improving the quality of grounded theory studies has been emphasized as critical to ensuring theoretical trustworthiness. Thus, the primary aim of the present study was to systematically review hearing loss research studies that have applied grounded theory methodology and assess the methodological quality of those grounded theory applications. Secondarily aims were to (i) explore how grounded theory methodology has been applied to investigate hearing loss, and (ii) use the findings of the review to develop a set of guidelines to aid the future high-quality application of grounded theory methodology to hearing loss research.

**Design:**

Original peer-reviewed studies applying grounded theory methodology and published in English were identified through systematic searches in 10 databases; Applied Social Sciences Index and Abstracts, British Nursing Index, Cumulative Index to Nursing and Allied Health Literature, EBSCO, Global Health, MEDLINE (OvidSP), PsycINFO, PubMed, Scopus, and Web of Science. The quality of studies was assessed according to 12 grounded theory principles using the Guideline for Reporting, Evaluating, and applying the core principles of Grounded Theory studies (GUREGT) tool. Data were analyzed using qualitative inductive thematic analysis.

**Results:**

After the removal of duplicates, 155 articles were retrieved. Of those, 39 met the criteria for inclusion in the systematic review. An increase in the adoption of grounded theory methodology to investigate hearing loss was identified with the number of published studies tripling in the last 5 years. Critical appraisal using the GUREGT tool identified four studies as high-quality. Most included studies were of moderate study quality (n = 25), and 10 were classified as being of low study quality. Using inductive thematic analysis, the included studies investigated one of four areas relating to hearing loss: (a) Living with hearing loss, (b) Identity and hearing loss, (c) Coping strategies for hearing loss, and (d) Audiological counseling and rehabilitation. Analysis also identified four main grounded theory factors frequently overlooked in hearing loss research: the different schools of grounded theory, sampling strategy, sample size, and the depth of grounded theory application.

**Conclusions:**

Use of grounded theory methodology is increasing at a rapid rate in hearing loss research. Despite this, studies conducted in the field to date do not meet and apply the full spectrum of grounded theory principles, as outlined by the GUREGT tool. To improve methodological rigor in future studies using grounded theory, we propose a set of guidelines that address the most commonly overlooked methodological considerations in hearing loss studies to date. The guidelines are designed to aid researchers to achieve high methodological quality in any field, improve qualitative rigor, and promote theoretical credibility.

## INTRODUCTION

Hearing loss is the third most common condition affecting the global population (World Health Organization, & Public Health Agency of Canada 2005; Bernell & Howard 2016; Vos et al. 2016; Scinicariello et al. 2019). It impacts 12 million people in the UK (RNID 2023), and 466 million people worldwide (Chadha et al. 2021). The number of people with hearing loss (PHL) is expected to double to 900 million by 2050 (World Health Organization 2018). Qualitative methodologies have been increasingly applied within the hearing loss field (Knudsen et al. 2012), largely due to the psychosocial impacts of hearing loss, which include communication difficulties (Demorest & Erdman 1987; Pryce et al. 2016), effects on intimate relationships (Barker et al. 2017) and work (Jennings & Shaw 2008), experiences of social isolation (Mick et al. 2014; Heffernan et al. 2016), depression (Hallam et al. 2006) anxiety (Gomaa et al. 2014; Contrera et al. 2017), and lower overall quality of life (Dalton et al. 2003; Nordvik et al. 2018; Punch et al. 2019). Such research has enhanced understanding of the impacts of living with hearing loss and aided development of patient-centered accommodations for PHL and their communication partners (CP).

The rise in qualitative studies in the field of hearing loss is largely due to an enhanced recognition of the value and insight they enable. Notably, this increase in popularity facilitated qualitative methods becoming more accepted and trusted within the field (Knudsen et al. 2012). Despite the usefulness of qualitative methods, quality of the work stems from the authors understanding and embracement of the appropriate research philosophy, which differs according to the qualitative methodology they choose (Creswell & Miller 2000). Particularly within scientific fields, including audiology, many researchers adopt a positivist philosophy, which focuses on objective and scientific standpoints. This can lead to decreased flexibility and subjectivity, which are central to interpretive philosophy and are processes that underpin qualitative research (Knudsen et al. 2012). Therefore, the aim of this systematic review was to provide insight into the state of applications of qualitative research in the field of hearing loss.

There are five main qualitative methodologies used to investigate human perspectives and behaviors. These include case studies, phenomenology, narratives, ethnography, and grounded theory. Grounded theory has been used in hearing loss research for the last two decades (Knudsen et al. 2012). It is used when an in-depth theory is needed to establish new understandings regarding a specific phenomenon (McCann & Polacsek 2018). The methodology is used to create novel theories rather than test existing ones (Glaser & Strauss 1965). The theories formed using grounded theory methodology exclusively stem from within the data, therefore the methodology acts as a thorough and substantiated approach for effectively exploring and explaining phenomena (Charmaz 2006; Glaser et al. 1968). Grounded theory incorporates popular analysis techniques used by qualitative researchers across disciplines (Gibbs 2012). It specifically involves the rigorous exploration of data in an open-minded, efficient, and systematic approach for forming a novel theory (Glaser et al. 1968; Charmaz 2006; Thornberg et al. 2014).

Glaser & Strauss 1967 established grounded theory methodology in 1967 after observational research investigating experiences of terminally ill patients was limited to descriptive insights. They identified a need for a systematic methodology that provides both descriptions and informative explanations of the processes under investigation (Glaser et al. 1968; Charmaz 2006; Morse et al. 2016). After creating the original school of grounded theory, Glaser and Strauss’ union dissolved due to differences in grounded theory approaches (Charmaz 2008b; Kenny & Fourie 2015). Glaser maintained the original philosophical stance of classic grounded theory, which aimed to discover an objective truth using systematic methods, now renamed as the Glaserian school (Glaser et al. 1968; Glaser & Strauss 1999; Charmaz 2008b; Kenny & Fourie 2015). This school suggests that a researcher can successfully remove their influence from an investigation and be able to achieve objective truths using constant comparative analysis and substantive coding (Glaser 2007).

Strauss formed the Straussian school of grounded theory, adopting symbolic interactionist philosophy, which emphasizes that knowledge is established through social interactions and communication (Strauss & Corbin 1997; Glaser 2005). It suggests that the theory formed, although able to achieve validity, is influenced by the researcher’s own biased interpretations (Annells 1997), and that establishing one generalizable truth is not possible as it is a subjective process (Corbin & Strauss 1990; Strauss & Corbin 1997). Therefore, to ensure the formation of a valid theory, a prescriptive approach to analysis (open, selective, axial coding, and matrix building) should be followed. Theoretical sensitivity, where the researcher is transparent in the theory formation process (Glaser & Holton 2004; Thornberg 2012), must also be established through reflexivity and reflections (Hall & Callery 2001).

Charmaz (2006), a student of Glaser and Strauss, evolved the methodology for a third time, maintaining principles from both theory founders with a new emphasis on constructivism. This suggests that reality is socially constructed by individuals within social contexts and through shared experiences with others (Charmaz 2006, 2008a). Therefore, the constructivist school outlines that a theory is mutually constructed through interactions between the researcher and the research subject, acknowledging the influence the researcher has during theory formation (Charmaz 2006, 2008a). Unlike the Straussian school, the constructivist school emphasizes the importance of fluidity during theory formation, suggesting that rigid analytic coding can hinder researchers from fully engaging with the data (Charmaz 2008a; Kenny & Fourie 2015). For a comparison of the three schools see Supplemental Digital Content 1, http://links.lww.com/EANDH/B279.

The need to evaluate the quality of grounded theory methodology within studies has been emphasized since its creation (Glaser et al. 1968) and since by researchers across disciplines (Urquhart et al. 2010), such as physiology (Hutchison et al. 2011; Ali et al. 2019), psychology (Weed 2009), nursing (Lazenbatt & Elliott 2005; McCann & Polacsek 2018), business and management (Douglas 2003; Christiansen 2011), and dental medicine (Sbaraini et al. 2011). Assessing the quality of grounded theory methodology is essential for ensuring that the application of the core principles remains consistent across the three grounded theory schools (Timonen et al. 2018; McCann & Polacsek 2018). This is particularly important given that a lack of consistency in the application of grounded theory methodology has been reported in healthcare research (Hussein et al. 2014; Timonen et al. 2018). This consequentially decreases the value and trustworthiness of emerging theories from the research and can lead to them being classified as unreliable (Morse et al. 2016; Berthelsen et al. 2018; Timonen et al. 2018).

Chronic illness research is the field each grounded theory school founder specialized in (Glaser and Strauss (1965), Corbin and Strauss (1985), and Charmaz (1990)), and has thus seen extensive applications and evaluations of grounded theory aiming to ensure methodological quality (Charmaz 1990; Baker & Stern 1993; da Silva Barreto et al. 2018). Charmaz identified grounded theory methodology as the most effective for studying chronic conditions, as it provides in-depth insights of the lived experiences, everyday impacts, opinions, and feelings of living with long-term conditions in different situations (Charmaz 1983, 1990; Charmaz & Belgrave 2001; Belgrave & Charmaz 2014).

In 2018, the first Guideline for Reporting, Evaluating, and applying the core principles of Grounded Theory studies (GUREGT) was created (Berthelsen et al. 2018). GUREGT is used to assess the quality of a grounded theory study by evaluating the depth of applications of the 12 core principles of grounded theory. The main GUREGT components are (1) Study aim: study should aim for theory formation; (2) Philosophical framework: study should adopt the correct philosophical framework based on the school of grounded theory followed; (3) The researcher’s role: study should address the researcher’s role through reflexivity; (4) Data collection: study should simultaneously collect and analyze data; (5) Memos: study should make memos throughout the research process; (6) Sampling procedures: studies should apply theoretical sampling, in which significant samples and concepts are pursued and explored further; (7) Theoretical saturation: studies should reach theoretical saturation, that is, no new information is being attained through data collection; (8) Analysis and coding: study should apply the appropriate analysis and coding techniques based on the school adopted; (9) Review of literature: study should either avoid or initially review the literature based on school followed; (10) Results/the theory: study should clearly and fully outline the results/theory developed; (11) Discussion: study should discuss key links between theory components and established literature; (12) Evaluation criteria: study should apply the appropriate evaluation criteria to evaluate the theory formed and establish its overall trustworthiness/validity. See Supplemental Digital Content 2, http://links.lww.com/EANDH/B280, for the GUREGT tool containing more specific details and how each principle is evaluated.

Despite hearing loss also being a chronic condition, previous systematic reviews that have assessed the quality of grounded theory applications in this field have excluded hearing loss studies (Conrad 1990; Baker & Stern 1993; da Silva Barreto et al. 2018). It has also been recommended for researchers in the field of hearing loss to ensure methodological quality of their studies (Knudsen et al. 2012), and avoid creating misinformed or poorly constructed theories that are misleading and fail to enrich existing knowledge (Dellve et al. 2002; Hallberg 2006; Meston & Ng 2012). Therefore, this systematic review is the first to assess the methodological quality of grounded theory studies within hearing loss research, informing future applications within the field. The primary aim of this review was to critically assess the methodological quality of grounded theory applications in hearing loss research using the GUREGT tool. Secondary aims were to (i) describe how grounded theory methodology has been applied to investigate hearing loss, and (ii) produce recommendations to guide researchers investigating hearing loss using grounded theory methodology to maximize future research quality.

## MATERIALS AND METHODS

This systematic review was conducted and reported in compliance with the Preferred Reporting Items for Systematic Reviews and Meta-Analysis checklist (Moher et al. 2015). The aim of this systematic review was not to synthesize the findings from the included studies. Rather, the aim of this review was to critically review the methodological completeness of the included studies. We only include a summary of the results from each review to offer context to our findings. The systematic review protocol was preregistered (PROSPERO: CRD42019134197) and published in a peer-reviewed journal (Ali et al. 2020).

### Search Strategy

Peer-reviewed journal articles were identified through searching ten databases: Applied Social Sciences Index and Abstracts (1987–current), British Nursing Index (1994–current), Cumulative Index to Nursing and Allied Health Literature (1961–current), EBSCO (1944–current), Global Health (OvidSP database, 1973–current), MEDLINE (Ovid, In-Process & Other Non-Indexed Citations, 1946–current), PsycINFO (1800s–current), PubMed (1996–current), Scopus (1983–current), and Web of Science (1899–current). Google Scholar was also used for forward citation tracking. A second search was conducted in March 2020, while the final search was conducted in August 2021 (see Supplemental Digital Content 3, http://links.lww.com/EANDH/B281, for Supplemental Search Strategy).

### Study Selection

A standardized approach was taken when assessing the eligibility of search results. The screening of titles and abstracts was performed independently by two reviewers (D.J.H.; Y.H.K.A.), and differences were resolved through discussions with a third reviewer (H.H.). Full text screening was also performed independently (H.H.; Y.H.K.A.) and differences were resolved through discussion between the two researchers.

**Fig. 1.**
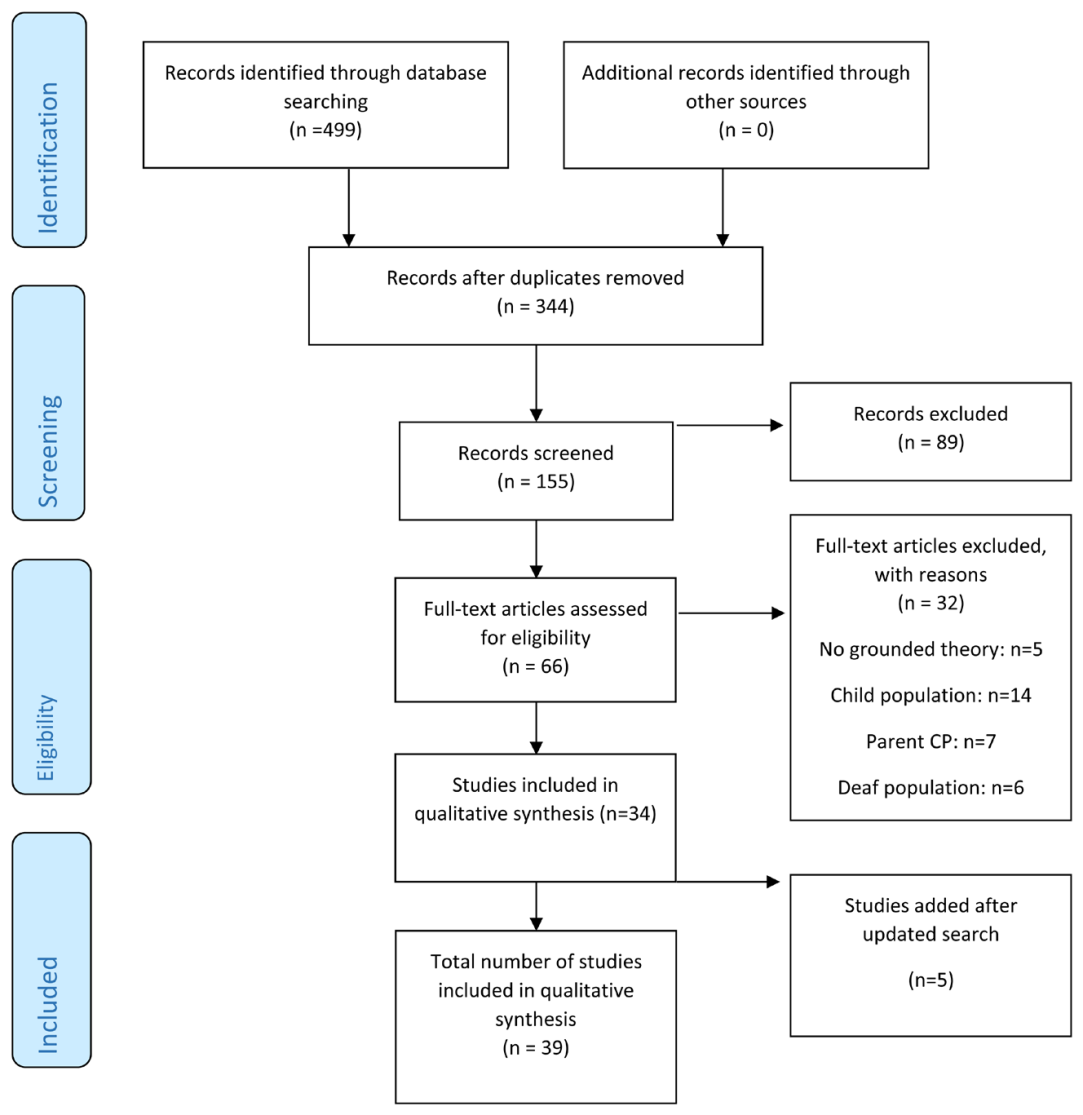
The PRISMA flow diagram of the study identification, screening, eligibility, and inclusion process within the systematic search of studies investigating hearing loss using grounded theory. PRISMA, Preferred Reporting Items for Systematic Reviews and Meta-Analysis.

For inclusion, studies had to have used grounded theory methodology while citing appropriate references relating to the methodological approach. Methodology: studies were included if they used grounded theory as either the main methodology, or as a secondary methodology embedded within overarching qualitative approaches such as phenomenology, ethnography, case studies, or narratives. Studies had to explicitly refer to using grounded theory during data collection and/or analysis for inclusion. This was assessed during full text screening. Studies that did not state using grounded theory were excluded. PhD theses that had been peer reviewed and met the inclusion criteria were also included in the systematic search. Study design: qualitative studies, or mixed methods studies using both qualitative and quantitative methods while applying grounded theory, were included. Purely quantitative studies that did not use grounded theory were excluded. Data format: Gray literature such as conference abstracts, book chapters, case reports, practice guidelines, and studies reporting expert opinions were excluded. This was due to gray literature not reporting primary research studies (e.g., book chapters, expert opinion pieces), which thus lack sufficient detail to meet inclusion criteria or to conduct a detailed evaluation of the study (e.g., conference abstracts) (Adams et al. 2017). Date of publication: studies published before 1967, the year grounded theory was first introduced (Glaser et al. 1968) were excluded. Language: Only studies published in English were included. Data screening and detection of duplicate studies were conducted using Covidence. See Figure 1 for the study filtration process based on the inclusion and exclusion criteria.

### Data Extraction

Data were extracted independently by two reviewers (H.M., Y.H.K.A.) into excel (Supplemental Digital Content 4, http://links.lww.com/EANDH/B282). Only exact information was extracted, and if the appropriate data had not been reported, it was documented and written as “not stated.” Any variance in the data extracted was discussed between reviewers until consensus was reached.

The following data were extracted:

i. Article characteristics include authors, title, year of publication, journal, funding, conflicts of interest, and ethical approval.
ii. Aims and objectives and type of hearing loss being investigated.
iii. Population information includes general characteristics, type of participant (i.e., PHL, CP, audiologists, healthcare practitioners such as audiologists, General Practitioners, Ear, Nose and Throat specialists, and hearing therapists), hearing loss characteristics (i.e., severity, years of hearing loss [HL], device used). Overall sample size.
iv. Study design and methodology, school of grounded theory followed.
v. Data collection.
vi. Key findings.
vii. Attempts to establish qualitative rigor and trustworthiness, limitations.
viii. Advantages and disadvantages of grounded theory.
ix. Conclusions and recommendations.

### Study Assessment

The GUREGT tool (Berthelsen et al. 2018) was used to determine methodological quality of the 39 included studies. Despite developing separately, all schools of grounded theory methodology share central grounded theory principles (Timonen et al. 2018), although applied differently (Smith & Biley 1997; Hutchison et al. 2011; Morse et al. 2016). These principles have been identified as imperative for any research using the methodology for the formation of trustworthy and accurate new theories (Charmaz 2006; Hutchison et al. 2011; Ali et al. 2019; Timonen et al. 2018).

The GUREGT tool was utilized due to its comprehensive approach and allowing for a methodological analysis of all included studies. GUREGT was developed to promote rigor and help maintain theoretical sensitivity and was the first specific method to evaluate grounded theory research. It enables researchers to identify and report how well-grounded theory principles were applied in a study while identifying any missing information. An issue faced during the study assessment was that some included studies did not specify which school of grounded theory they followed. Therefore, if a study did not state which grounded theory school they followed, the reference list and analysis techniques within the article were reviewed. Studies that were vague and did not include details of a specific techniques or relevant references were categorized as having followed the classical school.

Studies were independently analyzed by four reviewers (H.M., D.J.H., D.C., Y.H.K.A.). To increase fidelity of the tool and standardize the comparison of study quality, a scoring system was introduced to the GUREGT tool. The total scoring system in the present article was to aid in the clear categorization of each study, and transparently reflect how the level of adherence to grounded theory principles affected the overall methodological score of each study. Each item was assessed and given a score between 0 and 2. Scores of 0 implied the criteria for that item were not met, a score of 1 suggested the criteria were met to some extent and a score of 2 was given when the item criteria were satisfied. An overall score was then calculated and as some items included multiple elements, the highest score a paper could receive was 50. The number 50 is a direct reflection of the number of grounded theory methodological considerations established in the GUREGT tool when they are fully met. There are 25 subitems on the GUREGT scale which can include two considerations in one subitem for example, “*Is the selection of participants guided by theoretical sampling?*^1^ *How?*^2^” For this subitem, the reviewer examined the two parts of this question and scored accordingly, while also providing evidence for the score. If the study under review performed theoretical sampling and described how this was conducted, the subitem received a score of 2 for meeting both considerations. If theoretical sampling was stated to have been conducted but no detail was available to explain how it was conducted, a score of 1 was given. If theoretical sampling was not performed at all on this item, a score of 0 was given. This detailed process aided the reviewers to account for the completeness of reporting of each component of the subitems and ensured avoiding overlooking the multiple components under investigation grouped under one item. In addition to a score, some items required an explanation as to why and how the item was met. If there were discrepancies in the scoring system, a discussion was held between the second reviewer and the lead author, Y.H.K.A., until a final consensus was reached.

### Category Analysis

Category analysis was conducted to establish some descriptive characteristics of included studies. The frequency of grounded theory application in hearing loss research was established by reviewing the date of publication of each study, to understand the level at which grounded theory was used in hearing loss research. Studies were also categorized according to the area of hearing loss it specifically researched, to establish the research topics within hearing loss research that are commonly investigated using grounded theory.

### Thematic Analyses

Two researchers separately performed thematic analysis of the data (Y.H.K.A.; N.W.). Inductive thematic analysis as established by Braun and Clarke, was used as the primary analysis technique to code, thematize, and group themes relating to applications of grounded theory methodology (Braun & Clarke 2014; Clarke & Braun 2017). Line-by-line coding was applied by identifying the initial codes that related to grounded theory usage. These initial codes were then developed into more focused codes which identified recurrent interconnections between the initial codes. Categories were then formed from grouping focused codes, based on relevance and similarity. A higher-level categorization was then performed to identify specific themes based on significance where the most influential processes were established. The aims of extracted studies were analyzed to establish the overall areas of hearing loss that were mainly investigated using grounded theory. Multiple meetings were held between Y.H.K.A. and N.W. to compare analysis, the formation of codes and themes, with 100% agreement on the final themes being achieved.

### Findings

#### Descriptive Characteristics

Initial searches in June 2019 identified 499 records. After the removal of 344 duplicates 155 titles and abstracts were screened for potential eligibility. Following the screening, 93 records were excluded and the remaining 62 were subjected to full text review. All 62 records were read in detail and scrutinized against the established inclusion/exclusion criteria to determine eligibility. A further 28 records were excluded at the full text stage. Thirty-four records met the final inclusion criteria after the initial search in June 2019. An updated search was conducted in March 2020, resulting in the identification of three new studies, and a further updated search was conducted in August 2021, where two new studies were identified (Dunsmore et al. 2020; Koerber et al. 2022) giving; 39 records for inclusion in this review.

Of the 39 studies, 31 used only grounded theory methodology, whereas eight combined grounded theory with other qualitative research methods, and three were mixed methods studies. Data were collected using interviews in 33 of the reported studies, with 32 studies adopting a semi-structured approach and one adopting a structured approach, while six reported studies used focus groups. Observational methods were used as a secondary method of data collection in three of the included studies. All the included studies were peer-reviewed research articles.

Publication dates for included studies ranged from 1991 to 2021. From 1991 to 1995, four studies were published, and between 1996 and 2000, two studies were published. The number of published studies at the start of the 21st century (2001 to 2005) increased to five, and a further six studies were published between 2006 and 2010. While only five studies were published between 2011 and 2015; this number tripled to 17 studies published between 2016 and 2021.

**Fig. 2.**
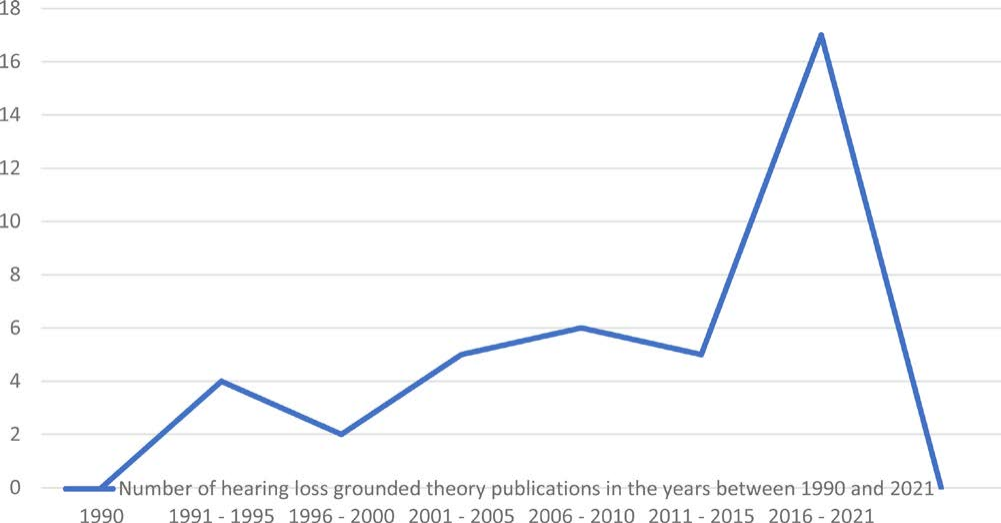
Showcasing the increasing trend of adopting grounded theory methodology in hearing loss research

These publication dates display a significant increase in the adoption of grounded theory methodology for investigating hearing loss in adults across the span of 30 years (Fig. 2).

The 39 studies included a total of 816 participants, with a mean of 20.9 (SD = 28.6), and mode of 10. Six out of the 39 studies had a sample size of 10 participants. Sample size varied from 1 to 168. Of all 39 studies, three did not report the gender of participants (Shaw et al. 2013; Gallagher & Woodside 2018; Gfeller et al. 2019). Of the 36 studies that did report gender, 47.2% (385/816) were male, and 47.9% (391/816) were female, indicating a somewhat equal investigation and inclusion of both genders across the reviewed studies. Thirty-two out of the 31 studies reported age; the mean age of participants was 51.2 years (SD = 15.30).

Degrees of hearing loss investigated varied from mild to profound depending on the area of investigation and aims of research. Studies also focused on different characterizations of HL. These included profound HL (n = 14 including CI related studies); presbyacusis (n = 7); severe HL (n = 5); postlingual sensorineural HL (n = 2); bilateral HL (n = 1); noise induced HL (n = 2); and acquired profound HL (n = 1). A significant number of studies focused on cochlear implant users and the process of adapting to their device (n = 7). Sample populations mainly consisted of three groups: PHL, CP, and healthcare practitioners. Most studies investigated only PHL (n = 29); two studies investigated CP (spouses/family carers of PHL), and four studies had a mix of PHL and CP samples. One study included all three groups of PHL, CP, and audiologist. Last, three studies only included health practitioners; these were audiologists, vocational rehabilitation therapists, and occupational therapists. Supplemental Digital Content 5, http://links.lww.com/EANDH/B283, provides a full description of included studies and their application of grounded theory principles or lack thereof.

### Aspects of Hearing Loss Investigated

**Fig. 3.**
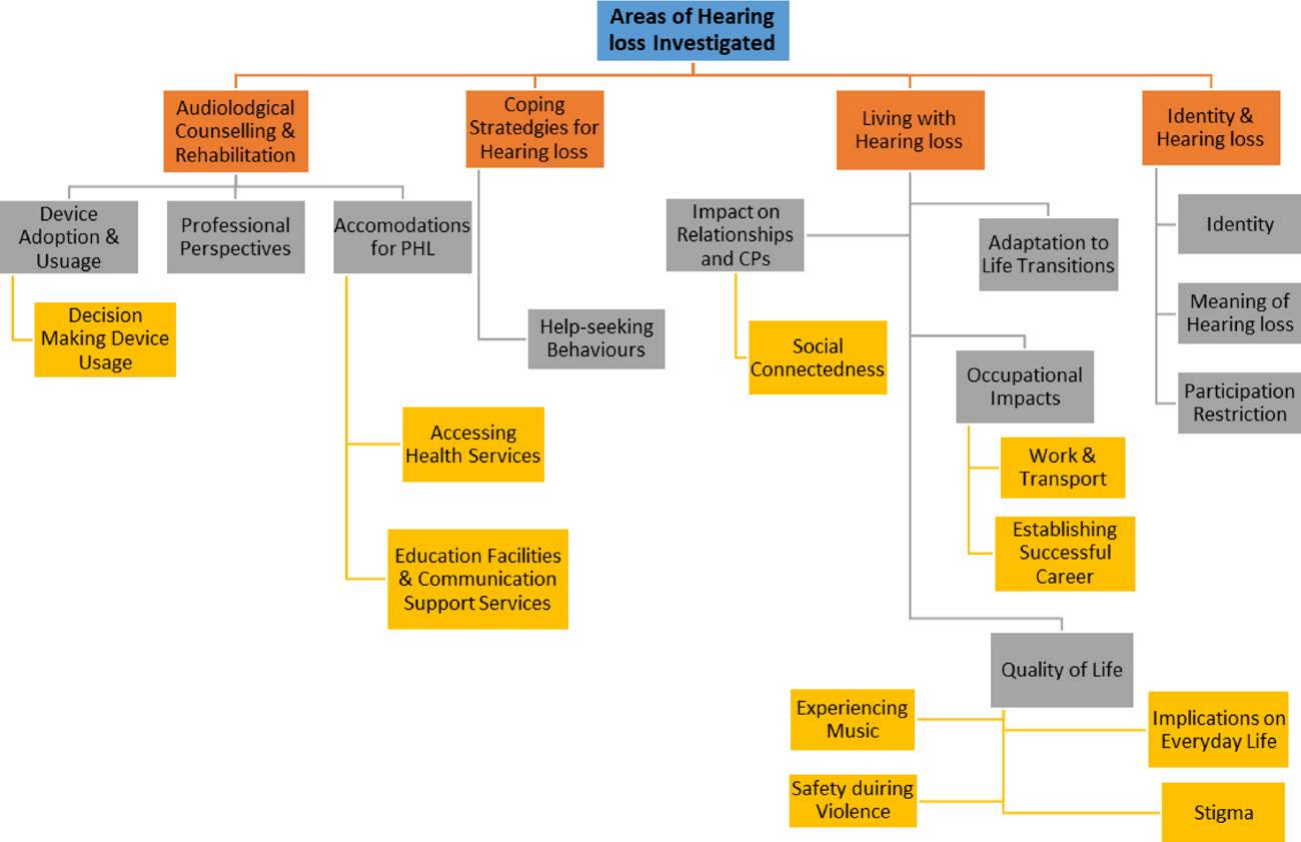
Areas of hearing loss investigated with themes and subthemes.

Four main themes and accompanying subthemes were identified (Fig. 3). The most often explored theme was “Living with Hearing loss” which accounted for the area of investigation of 17/39 studies. Subthemes included the impact of hearing loss on relationships and CP, investigating how hearing loss impacted the quality of relationships between a PHL and their significant other (n = 5); occupational impacts (n = 3); adaptation to life transitions (n = 1); implications on everyday life (n = 1); work and transport (n = 1); social connectedness (n = 1); quality of life (n = 1); establishing successful career (n = 1); experiencing music (n = 1); safety during violence (n = 1); stigma (n = 1). “Audiological counseling and rehabilitation” were the second most researched area identified, with 11/39 studies investigating within this topic. Subthemes including device adoption and usage (n = 3) which mainly looked at hearing aid and cochlear implant devices; decision-making of hearing device usage (n = 2), and “Professional Perspectives” as some studies focused on acquiring professional expertise regarding hearing loss considerations (n = 4); accommodations for PHL, education facilities, and communication support services (n = 1); and accessing health services with hearing loss (n = 1). The area of “Identity and hearing loss” was the third most frequently investigated theme with 6 of the 39 included studies. Four studies investigated identity alone, while the others investigated the: meaning of hearing loss (n = 1); and participation restrictions due to hearing loss (n = 1). Last, *“*Coping strategies for hearing loss” were the least explored theme, being investigated in 5/39 studies. Three coping studies investigating coping strategies were conducted by Hallberg (1991), the first researcher to apply grounded theory to investigate hearing loss in 1991. “Patterns in help-seeking behaviors for hearing loss” was a subtheme, being investigated twice (n = 2). Overall, grounded theory was applied to explore a diverse range of topics regarding the hearing loss experience, offering unprecedented insights into the experience of living with hearing loss, its impact on patients, their close family and friends, and the expert opinion of various healthcare practitioners.

### Methodological Quality

#### Quality Appraisal Performance

Three overall sorting categories were created based on the level of application of grounded theory principles, as identified by the GUREGT tool. Please see Supplemental Digital Content 5, http://links.lww.com/EANDH/B283, for the detailed methodological quality appraisal of each study which contains how they scored against each of the 50 grounded theory considerations. A study was categorized as low quality if it scored 0 to 19/50, moderate quality when it scored 20 to 39/50, or high quality when it scored 40+/50. Ten studies (n = 10) scored between 0–19 out of 50 using the GUREGT quality appraisal tool, as such these were classified as having low quality as they reached less than half. Most studies had moderate quality (n = 25) scoring between 20 and 39/50. Only four studies scored 40+/50, achieving a high-quality classification (n = 3). The highest GUREGT score given was 47/50 for the study by Martin (2010). This PhD thesis applied all central elements of grounded theory principles, and only lost three lost marks due to not justifying the use of quotes, and not identifying the basic social process before conducting focused coding. The second highest scoring record was also a PhD thesis investigating hearing loss identity authored by Dorminy (2014). It scored 42/50; losing eight points because a crucial grounded theory process, namely theoretical sampling, was not applied. The third study with a high score of 40/50 was also a PhD thesis by Hughes (2001) which despite using theoretical sampling and evaluation criteria, did not state their philosophical stance nor identify clear study aims.

The lowest scoring record was by McRackan et al. (2017), scoring 9/50. Many grounded theory principles were not met, including not applying theoretical sampling, constant comparative analysis and not discussing or evaluating a theory. Crucially, this study did not use grounded theory to create a new theory, and rather applied the methodology as a tool for analysis.

#### Evaluation of Resultant Grounded Theory

The study with the highest GUREGT score that was not a PhD thesis was by Hughes et al. (2018), investigating social connectedness in cochlear implant users, receiving 36/50. It applied most principles of grounded theory comprehensively and was transparent in reporting how it applied the methodology, identifying all methodological and analytical processes undertaken. It applied most grounded theory principles such as theoretical sampling, making memos, correct analysis techniques, constant comparative analysis, and even applied reflexivity on the research team’s background and expertise. The points lost were for not applying evaluative criteria to test the trustworthiness of the model developed. However, this is a common issue across most grounded theory studies included in this review as only seven of the 39 studies discussed and applied evaluative criteria. Some studies that did identify the recommended evaluative criteria for their school, failed to use them to critique the theory they developed (n = 4).

### Overlooked Principles of Grounded Theory

Data extraction and quality appraisals were also qualitatively analyzed using initial and focused coding. The following themes were detected as the most significant principles that impacted the overall perceived quality and score of grounded theory studies using the GUREGT tool. Four key principles of grounded theory were commonly overlooked and significantly impacted the overall quality of the methodological application. These were the different schools of grounded theory; the sample strategy, sample size; and the depth of grounded theory application.

#### The Different Schools of Grounded Theory

Only 15 studies clearly stated which school of grounded theory they followed. This is problematic as the GUREGT tool is based on the three different grounded theory schools and has a tailored set of evaluative criteria for each school. To overcome this, we examined the references and analysis techniques stated in the remaining studies to determine which school was likely to have been followed. This was successful for 20 studies which could be classified based on the methodological implications in the paper. However, four studies had no clear indication or reference to which grounded theory approach was applied. Overall, the schools of grounded theory followed differed across studies, with the most followed school being the constructivist school (n = 18) by Charmaz (2006), followed by the pragmatist school founded by Strauss and Corbin (1997) (n = 14), and the Glaserian (classical) school had the least application (n = 7).

Most records lacked detail of the grounded theory principles they followed, which is indicated in the difficulty of classifying each study’s grounded theory approach. The principles most successfully applied were mainly the core grounded theory principles that are central in all schools, such as constant comparative analysis, simultaneous data collection and analysis, and memoing. A general grounded theory approach was a common theme during analysis, and only a few studies engaged with the philosophical underpinnings and unique grounded theory tools of the specific grounded theory school it followed. Evidently, there was some confusion regarding the differences between schools as one study (Fitzpatrick & Schramm 2006) stated that they were following the principles of the constructivist school, while applying the principles of another school (the pragmatist school).

#### Sample Strategy

There were inconsistent applications of theoretical sampling across studies. Only 14 studies applied theoretical sampling, which is a core grounded theory principle. The remaining studies applied either initial sampling, opportunity sampling, or purposive sampling only at one instance (n = 25). Theoretical sampling is measured at three different stages on the GUREGT tool, therefore studies that did not apply it were impacted with a significantly decreased score.

#### Sample Size

Despite not being stated as a core grounded theory principle within the GUREGT tool, there is an agreed recommended sample size for grounded theory studies of at least 25 participants (Thomson 2010). This is to ensure insight from a range of perspectives and the development of a comprehensive and rich theory. Only nine studies had a sample of 25 or more participants, meaning 30 studies did not meet this grounded theory recommendation. No justifications for low numbers of participants recruited were given. Thus, studies with a sample size below n = 25 impairs the perceived comprehensiveness of the resultant theory which is crucial for the trustworthiness of any grounded theory developed.

#### Depth of Grounded Theory Application

The main utilization of grounded theory is for creating novel theories. Despite this, eight studies did not create a novel theory, and only used grounded theory within its analysis for thematic generation rather than theory generation. Therefore, 31 studies did produce a novel theory. Scoring low on the GUREGT tool, was consistent with the studies that did not fully apply the methodology to develop a novel theory. Uniquely, all studies applied grounded theory methodological techniques for data analysis (n = 39). This highlights grounded theory as a particularly useful analytical tool that qualitative researchers utilize for its thorough and systematic qualities.

## DISCUSSION

This systematic review has shown an increase in the adoption of grounded theory in hearing loss research, tripling within the last 5 years. However, most of the studies in the review scored below 30/50 on our GUREGT scale reflecting failure to report all core items listed on the GUREGT appraisal tool. Thus, the main issues that hindered the quality of the grounded theory studies reviewed will be discussed, and recommendations to overcome these issues will be identified to guide future researchers using the methodology in their field.

### Fluidity of Grounded Theory

Studies offered insight into which areas of hearing loss were commonly investigated using grounded theory. A number of diverse topics were investigated including the impacts of living with hearing loss on family relationships (Hallam et al. 2008), occupational health (Svinndal et al. 2020), assistive devices (Fitzpatrick & Leblanc 2010), and identity formation (Knudsen et al. 2012; Adler 2018). This confirms the proposed fluidity of the methodology praised by researchers as its main strength. Contemporary grounded theorists in varying health-related disciplines agree that the methodological flexibility offered by grounded theory has provided unprecedented insights in their disciplines. For example, grounded theory has been used to develop new policies regarding eating disorders (Faija et al. 2017), give a voice to silenced victims (Nova et al. 2018; Otake 2019), and shape the most common thematic analysis approach in qualitative research implemented across disciplines (Clarke & Braun 2017; Charmaz & Thornberg 2020). The range of topics investigated using grounded theory confirms the usefulness methodology for in-depth explorations of underlying social and psychological factors as maintained by grounded theorists (Thornberg et al. 2014; Chun Tie et al. 2019).

The significance of using grounded theory for hearing loss research has been in its enablement of identifying and forming the basis of new research areas within the field (Knudsen et al. 2012; Meston & Ng 2012). For example, the first development of a theory that identifies and explains the different coping styles PHL use to deal with their condition and how it impacts their auditory rehabilitation journey (Hallberg 1999) today acts as the basis of coping research within the field (Hricová 2018; Warringa et al. 2020). The findings from the current review reinforce the evident usefulness of grounded theory as a qualitative methodology for applications within hearing loss research and all health-related fields.

### A Theory Ungrounded

The quality of grounded theory was diminished by studies overlooking core principles that are essential for establishing the “grounded” nature of the methodology. Evidently, some studies used grounded theory solely as an analytical tool rather than a comprehensive methodological approach. The misuse of grounded theory has commonly been identified as a detrimental factor reducing the quality of studies within grounded theory methodological reviews, including engineering (Stol et al. 2016). A critical review of grounded theory applications in the field of exercise psychology identified that a substantial number of studies only used grounded theory as an analytical tool, leading the authors to hold a stern stance against this “cherry picking” approach (Hutchison et al. 2011). They explained that this inappropriate use of the methodology is “fundamentally flawed” as it fails to apply the basic principles of grounded theory and exploits the fluid nature of the methodology (Hutchison et al. 2011). This defies the consistency advocated by the founders of grounded theory (Charmaz 2006; Thornberg et al. 2014). Thus, selective application of grounded theory is discouraged in methodological recommendations for health research as the methodology was designed to inform all stages of a study: design, data collection, sampling, data analysis, and theory formation and evaluation (Charmaz 2006; Hutchison et al. 2011; Berthelsen et al. 2018).

To ensure the grounded nature of a qualitative study, researchers are recommended to embrace the qualitative philosophies advocated by their chosen methodology. Ward et al. (2015) identified the difficulties of researchers in health-related fields in transitioning from their scientific views and objective research philosophy (positivism) to the more subjective and interpretive worldview that is central to qualitative research (interpretivism). This systematic review emphasizes the importance of training and educating researchers in qualitative philosophy, from formal training and qualitative modules to discussions with qualitative researcher peers. Interpretive philosophical understandings must act as a background step before undertaking any research, as it is crucial for qualitative trustworthiness and rigor, and will influence each stage of a research project.

### Sampling Beyond Initial Recruitment

Due to the exhaustive building process of a novel theory, the need for a more thorough technique for sampling was identified at the outset of grounded theory construction (Glaser & Strauss 2006). To ensure a study was not limited by the initial recruited sample, and that sampling can continue as an iterative process directing theory explorations during the simultaneous data collection and analysis process, the notion of theoretical sampling was formed (Morse & Clark 2019). Theoretical sampling is a process by which focused explorations are made after identifying significant processes from the initial sample’s data. These insights are explored and refined by recruiting more participants with specific characteristics or expanding on specific processes in subsequent interviews (Charmaz 2006). Each grounded theory school stresses the importance of applying theoretical sampling, and the founders of the methodology identified it as a central process for increasing analytical power in any grounded theory (Glaser et al. 1968; Glaser & Strauss 2006). The current review found poor applications and reporting of theoretical sampling despite this being a fundamental methodological dimension; this indicates a common underestimation of the importance of the process in hearing loss research. Another systematic review investigating the application of theoretical sampling in nursing studies using grounded theory methodology found that 50% of the studies applied purposive sampling only, disregarding the use theoretical sampling (McCrae & Purssell 2016). Therefore, overlooking theoretical sampling as a central grounded theory process is a common and recurring issue impacting the quality of grounded theory studies in health research. To overcome this, McCrae and Purssell (2016) emphasized the need to establish systematic guidelines for studies to follow and ensure transparent reporting of their grounded theory process.

### Applying the Grounded Without the Theory

The main reason for using grounded theory methodology is to create a comprehensive novel theory (Glaser & Strauss 2014; Thornberg et al. 2014; Charmaz & Thornberg 2020). Despite this, we find that some studies only used grounded theory to analyze data and generate qualitative themes, and not to forge a theory. Nine out of the 39 studies did not include a novel theory in their study despite using grounded theory (Supplemental Digital Content 5, http://links.lww.com/EANDH/B283). This is reflective of limited methodological awareness during the study design stage, as the authors therefore may not have given much thought as to which methodology is the most suitable to adopt based on their study aims.

The results of this review showcase the increase in applying grounded theory as a methodology in the field of hearing loss research. Within grounded theory, data analysis is entirely dependent on the rigor of the data collection process. Quantitative researchers in the field of hearing loss newly adopting grounded theory may apply fewer data collection techniques, as not many methods rely on concrete data collection stages, which directly impact the data quality such as with grounded theory. To ensure adequate awareness of grounded theory process, knowledge or training of the methodology is needed before planning and implementing grounded theory studies.

A recent publication by Charmaz (2017) advocates for grounded theorists to develop methodological self-consciousness before the study design. This process involves researchers employing reflexivity throughout their research process, beginning with clear understandings of why grounded theory was specifically chosen as a methodology, which school is being followed, the philosophical underpinnings and the primary grounded theory principles, and how these subsequently shape and influence study design, data collection, analysis, and theory formation (Charmaz 2017; Charmaz & Thornberg 2020). Methodological self-consciousness is a central process for ensuring a rigorous and transparent application of grounded theory methodology and enhancing the quality of grounded theory methodological applications (Charmaz & Thornberg 2020). Therefore, the current review advocates the adoption of this practice throughout the research process as a tool to overcome low-quality grounded theory applications, and to enhance the trustworthiness of the resultant theories.

### Methodological Quality

The most frequently overlooked methodological factor that decreased the quality scores of studies in this review was to not evaluate grounded theory that was formed. Due to the comprehensive nature of grounded theory, the methodology has its own criteria to evaluate the quality of resultant theories which must undergo validation before other studies begin to build on their theoretical assumptions (Lazenbatt & Elliott 2005; Berthelsen et al. 2018). The evaluative criterion in grounded theory differs slightly based on the grounded theory school followed. A lack of understanding of the differences between grounded theory schools was found, with some studies following a different school’s evaluative criteria to the one identified. The classical school first emphasizes credibility of the researcher’s expertise, and establishment of a credible theory. Second is applicability, assessing how applicable the theory is to the field, similar to generalizability (Glaser et al. 1968). To judge methodological quality four criteria were identified: fit: the fittingness of theory as situated in the field, modifiability: how modifiable the theory is if new data arises, workability: how well the theory works to explain the phenomenon investigated, and relevance: how relevant the theory is for the population investigated (Smith & Biley 1997). The pragmatist school evaluates the quality of the data by considering empirical grounding of the theory and explanatory power, plausibility of the established theory and its potential value, and the adequacy of the research process (Corbin & Strauss 1990). Last, the constructivist school criteria involves four dimensions: usefulness, credibility, originality, and resonance of the theory (Charmaz 2006; Charmaz & Thornberg 2020). As seen in this review, there has been a lack of consistency in applying these evaluative criteria to establish the credibility and trustworthiness in grounded theory studies (Lomborg & Kirkevold 2003). Discarding the final recommended stage of grounded theory causes ambiguity regarding the applicability of the theory, and decreases the theory’s usefulness (Lomborg & Kirkevold 2003; Charmaz & Thornberg 2020). It is not clear why an essential and final stage of the theory building process is commonly overlooked; researchers outline that the time-consuming and intensive nature of grounded theory process can result in there being less time available to complete this further step (Miller 1995). Although this may indeed be a practical limitation of the methodology, a lack of grounded theory training and awareness of fundamental principles is evident and the major factor for all methodological downfalls discussed thus far.

### Advanced Grounded Theory Practice Needed

The many downfalls of grounded theory application in studies reviewed have shown limited grounded theory methodological depth and knowledge, with no study meeting all the grounded theory principles set. It has been agreed that, because of the fluid nature of the methodology, general guidelines are most suitable to evaluate grounded theory studies, and are essential for ensuring methodological quality (Charmaz & Thornberg 2020).

After the identification of the main downfalls researchers faced when conducting their studies, we propose guidelines consisting of 10 steps to aid researchers across disciplines achieve methodological excellence by designing and establishing a quality grounded theory study (Supplemental Digital Content 6, http://links.lww.com/EANDH/B284). These cover: 1. choosing the most appropriate grounded theory methodology, 2. employing methodological self-consciousness, 3. outlining the philosophical framework, 4. following the appropriate school of grounded theory, 5. in-depth application of methodology, 6. ensuring application of all core grounded theory principles, 7. appropriate sample size, 8. qualitative rigour, 9. evaluation of theory, and 10. transparency in publication. Most quality appraisal tools such as GUREGT, are referred to and used after the start, or even completion, of conducting a grounded theory study. It is then used to assess, rather than inform the development of a grounded theory study, with opportunities for researchers to improve their application of grounded theory principles being limited or nonexistent after their study has concluded. Our developed guidelines therefore aim to aid researchers from the point of study conception, to guide grounded theory considerations throughout the research process.

## LIMITATIONS

This systematic review used the GUREGT quality appraisal tool (Berthelsen et al. 2018) to evaluate the applications of included studies. GUREGT was thorough in providing a comprehensive and detailed listing of all main grounded theory principles and methodological considerations. However, the overall appraisal of some studies may not be fully representative of methodological quality due to the “all or none” nature of GUREGT, which could have overlooked some aspects of grounded theory. For example, some studies were comprehensive in their use of grounded theory methodology, while also applying other qualitative methodological techniques. The qualitative process followed by high quality studies in the review includes triangulation, crystallization, member-checking, each of which enhances the quality of a qualitative study, and in turn the quality of the grounded theory study if incorporated. However, the GUREGT tool offers no space to consider these factors which would increase the quality rating of a qualitative study in general (Rolfe 2006). Therefore, the use of a qualitative critical appraisal tool in addition to the GUREGT tool may offer greater fairness for future grounded theory methodological reviews.

To interpret the tool more quantitatively, we introduced a scoring, through which each item was assessed and given a score between 0 and 2, with 0 implying the criteria for that item was not met, 1 implying the criteria was met to some extent, and 2 when the item criteria were satisfied. The GUREGT tool mainly consists of open questions, which made it difficult to concisely compare ratings. Therefore, this scoring system was created to standardize criteria across the four researchers conducting the critical appraisal. Despite introducing this system, it did not address all issues as some questions within the GUREGT tool still required open interpretation. Despite citing evidence from studies to substantiate scores given and agreeing on all scores as a team, lacking specific detail on how to perform the appraisal for open-ended items created some lack of conciseness. Therefore, the current review recommends adding explicit detail in qualitative appraisal tools on how to assess subjective items and how to compare ratings, to aid in assessing grounding theory applications more consistently.

Another limitation was the inclusion of PhD theses within the systematic review. This may have skewed the critical appraisal finings as the top four scoring studies were either a PhD thesis (n = 3) or a paper resultant from completing a PhD (n = 1). The thoroughness and availability of large word counts for a thesis may account for the highest GUREGT scores as the comprehensive nature of a PhD thesis allows for more detail and transparency regarding the research process, as researchers are also assessed on this basis. Therefore, the quality appraisal results may not be representative, and grounded theory applications may be even lower than reported had these studies been excluded.

## CONCLUSION

The findings of this systematic review offer several implications for the utilization of grounded theory as a methodology in hearing loss research. The review found that applications of grounded theory have become increasingly adopted as a methodology in hearing loss research, showcasing the evident importance of ensuring grounded theory quality for the development of trustworthy novel theories across a range of topics within hearing loss research. The main issue that undermined the rigor of reviewed studies was not applying grounded theory to create a new theory, and many studies that did not fully apply the principles would enable their study to be classified as grounded. These methodological factors included not applying theoretical sampling, having a small sample size, and selective applications of grounded theory as an analytic tool. The quality of grounded theory applications was mostly hindered by a failure to apply evaluative criteria to assess the trustworthiness of theories developed. A significant lack of thorough methodological application was identified, and a substantial need for greater awareness of grounded theory principles is evident. It is recommended that grounded theorists follow grounded theory guidelines in future studies to overcome these methodological downfalls. The 10-step guidelines have been established considering the findings of this review, to aid grounded theorists across disciplines to achieve methodological awareness and apply grounded theory principles throughout their study from conception to dissemination. Future research using the 10-step guidelines in this review could evaluate its usefulness in their studies, providing opportunities for future refinement. The value of grounded theory methodology with its fluid and thorough nature has become evident in this review. When applied rigorously, the potential of achieving significant insights in the field of hearing loss and health research is high.

## ACKNOWLEDGMENTS

The authors dedicate this article to the late Professor Kathy Charmaz, the developer of constructivist grounded theory (August 19, 1939 to July 27, 2020) and offer their sincere condolences. Her contributions to the field of chronic illness and reformations in grounded theory methodology will continue to inspire the current authors and many generations to come.

## Supplementary Material



## References

[R1] Adams R. J. Smart P. Huff A. S. (2017). Shades of grey: Guidelines for working with the grey literature in systematic reviews for management and organizational studies. Int J Manag Rev, 19, 432–454.

[R2] Adler J. M. (2018). Bringing the (disabled) body to personality psychology: A case study of Samantha. J Pers, 86, 803–824. 29222912 10.1111/jopy.12364

[R3] Ali N. May S. Grafton K. (2019). A systematic review of grounded theory studies in physiotherapy. Physiother Theory Pract, 35, 1139–1169. 29791261 10.1080/09593985.2018.1474403

[R4] Ali Y. H. K. Wright N. Charnock D. Henshaw H. Hoare D. (2020). Applications of qualitative grounded theory methodology to investigate hearing loss: Protocol for a qualitative systematic review. BMJ Open, 10, e033537. 10.1136/bmjopen-2019-033537 PMC7200034 32295772

[R5] Annells M. (1997). Grounded theory method, part I: Within the five moments of qualitative research. Nurs Inq, 4, 120–129. 9224048 10.1111/j.1440-1800.1997.tb00085.x

[R6] Baker C., & Stern P. N. (1993). Finding meaning in chronic illness as the key to self-care. Can J Nurs Res, 25, 23–36. 8118760

[R7] Barker A. B. Leighton P. Ferguson M. A. (2017). Coping together with hearing loss: A qualitative meta-synthesis of the psychosocial experiences of people with hearing loss and their communication partners. Int J Audiol, 56, 297–305. 28599604 10.1080/14992027.2017.1286695

[R8] Belgrave L. L., & Charmaz K. (2014). Studying illness and dying through constructivist grounded theory. In Van Brussel L., & Carpentier N. (Eds.), The Social Construction of Death (pp 34–51). Palgrave Macmillan. 10.1057/9781137391919_3

[R9] Bernell S., & Howard S. W. (2016). Use your words carefully: What is a chronic disease? Front Public Health, 4, 159–159. 27532034 10.3389/fpubh.2016.00159 PMC4969287

[R10] Berthelsen C. B. Grimshaw-Aagaard S. L. S. Hansen C. (2018). Developing a guideline for reporting and evaluating grounded theory research studies (GUREGT). Int J Health Sci, 6, 64–76.

[R11] Braun V., & Clarke V. (2014). What can “thematic analysis” offer health and wellbeing researchers? Int J Qual Stud Health Well-being, 9, 26152. 25326092 10.3402/qhw.v9.26152 PMC4201665

[R12] Chadha S. Kamenov K. Cieza A. (2021). The world report on hearing, 2021. Bull World Health Organ, 99, 242–242A. 33953438 10.2471/BLT.21.285643 PMC8085630

[R13] Charmaz K. (1983). Loss of self: A fundamental form of suffering in the chronically ill. Sociol Health Illn, 5, 168–195. 10261981 10.1111/1467-9566.ep10491512

[R14] Charmaz K. (1990). ‘Discovering’ chronic illness: Using grounded theory. Soc Sci Med, 30, 1161–1172. 2360052 10.1016/0277-9536(90)90256-r

[R15] Charmaz K. (2006). Constructing Grounded Theory: A Practical Guide Through Qualitative Analysis (1st ed.). Sage

[R16] Charmaz K. (2008a). Constructionism and the grounded theory method. In J. A. Holstein & J. F. Gubrium (Eds.), Handbook of Constructionist Research (pp. 397–412). Guilford.

[R17] Charmaz K. (2008b). Chapter 7 Grounded theory as an emergent method. In S. N. Hesse-Biber & P. Leavy (Eds.), Handbook of Emergent Methods. (pp. 155–168). Guildford.

[R18] Charmaz K. (2017). The power of constructivist grounded theory for critical inquiry. Qual Inq, 23, 34–45.

[R19] Charmaz K., & Belgrave L. (2001). Qualitative interviewing and grounded theory analysis. In J. Gubrium & J. Holstein (Eds.), The Handbook of Interview Research (pp. 675–694). Sage. 10.1177/0001699313496732.

[R20] Charmaz K., & Thornberg R. (2020). The pursuit of quality in grounded theory. Qual Res Psychol, 18, 305–327.

[R21] Christiansen O. (2011). Rethinking “quality” by classic grounded theory. Int J Qual Serv Sci, 3, 199–210.

[R22] Chun Tie Y. Birks M. Francis K. (2019). Grounded theory research: A design framework for novice researchers. SAGE Open Med, 7, 2050312118822927. 30637106 10.1177/2050312118822927 PMC6318722

[R23] Clarke V., & Braun V. (2017). Thematic analysis. J Posit Psychol, 12, 297–298.

[R24] Conrad P. (1990). Qualitative research on chronic illness: A commentary on method and conceptual development. Soc Sci Med, 30, 1257–1263. 2360060 10.1016/0277-9536(90)90266-u

[R25] Contrera K. J. Betz J. Deal J. Choi J. S. Ayonayon H. N. Harris T. Helzner E. Martin K. R. Mehta K. Pratt S. Rubin S. M. Satterfield S. Yaffe K. Simonsick E. M. Lin F. R.; Health ABC Study. (2017). Association of hearing impairment and anxiety in older adults. J Aging Health, 29, 172–184. 26916793 10.1177/0898264316634571 PMC5704938

[R26] Corbin J., & Strauss A. L. (1985). Managing chronic illness at home: Three lines of work. Qual Sociol, 8, 224–247.

[R27] Corbin J., & Strauss A. L. (1990). Grounded theory research: Procedures, canons, and evaluative criteria. Qual Sociol, 13, 3–21.

[R28] Creswell J. W., & Miller D. L. (2000). Determining validity in qualitative inquiry. Theory Pract, 39, 124–130.

[R29] da Silva Barreto M. Garcia-Vivar C. Marcon S. S. (2018). Methodological quality of Grounded Theory research with families living with chronic illness. Int J Africa Nurs Sci, 8, 14–22.

[R30] Dalton D. S. Cruickshanks K. J. Klein B. E. Klein R. Wiley T. L. Nondahl D. M. (2003). The impact of hearing loss on quality of life in older adults. Gerontologist, 43, 661–668. 14570962 10.1093/geront/43.5.661

[R31] Dellve L. Henning-Abrahamsson K. Trulsson U., & Hallberg L. R. (2002). Grounded theory in public health research. In L. R. M. Hallberg (Ed.), Qualitative Methods in Public Health Research (pp. 137–174). Studentlitterature.

[R32] Demorest M., & Erdman S. (1987). Development of the communication profile for the hearing impaired. J Speech Hear Disord, 52, 129–143. 3573744 10.1044/jshd.5202.129

[R33] Dorminy J. L. (2014). The experiences of non-signing deaf and hard-of-hearing students and their academic and social integration into a primarily signing deaf university environment (Order No. 3590754). ProQuest Dissertations & Theses A&I (1432725117). Retrieved from https://www.proquest.com/dissertations-theses/experiences-non-signing-deaf-hard-hearing/docview/1432725117/se-2

[R34] Douglas D. (2003). Grounded theories of management: A methodological review. Manage Res News, 26, 44–52.

[R35] Dunsmore M. E. Schneider J. McKenzie H. Gillespie J. A. (2020). The effort of caring: The caregivers’ perspective of dual sensory impairment. Front Educ (Lausanne), 5, 572201.

[R36] Faija C. L. Tierney S. Gooding P. A. Peters S. Fox J. R. (2017). The role of pride in women with anorexia nervosa: A grounded theory study. Psychol Psychother, 90, 567–585. 28467686 10.1111/papt.12125

[R37] Fitzpatrick E., & Leblanc S. (2010). Exploring the factors influencing discontinued hearing aid use in patients with unilateral cochlear implants. Trends Amplif, 14, 199–210. 21406420 10.1177/1084713810396511 PMC4111407

[R38] Fitzpatrick E., & Schramm D. (2006). Clinicians’ perceptions of cochlear implant benefits in adults with prelingual deafness. J Speech-Lang-Pathol Audiol, 30, 192–197.

[R39] Gallagher N. E., & Woodside J. V. (2018). Factors affecting hearing aid adoption and use: A qualitative study. J Am Acad Audiol, 29, 300–312. 29664724 10.3766/jaaa.16148

[R40] Gfeller K. Driscoll V. Schwalje A. (2019). Adult cochlear implant recipients’ perspectives on experiences with music in everyday life: A multifaceted and dynamic phenomenon. Front Neurosci, 13, 1229. 31824240 10.3389/fnins.2019.01229 PMC6882382

[R41] Gibbs G. R. (2012). Grounded theory, coding and computer-assisted analysis. In S. Becker A. Bryman, & H. Ferguson (Eds.), Understanding Research for Social Policy and Social Work: Themes, Methods and Approaches, Understanding Welfare: Social Issues, Policy and Practice Series (2nd ed., pp. 337–343). Policy Press. http://eprints.hud.ac.uk/id/eprint/13124/

[R42] Glaser B. G. (2005). The impact of symbolic interaction on grounded theory. The *Grounded Theory Review* (Vol. 4, pp. 1–22). Sociology Press.

[R43] Glaser B. G. (2007). Doing formal theory. The Sage Handbook of Grounded Theory (Part II), (pp. 97–113). SAGE Publications Ltd. 10.4135/9781848607941

[R44] Glaser B. G. & Strauss A. L. (1965). Awareness of Dying (1st ed.). Routledge. 10.4324/9781351327923

[R45] Glaser B. G. & Strauss A. L. (1999). Grounded Theory: Strategies for Qualitative Research (1st ed.). Routledge. 10.4324/9780203793206

[R46] Glaser B. G. & Strauss A. L. (2006). Theoretical sampling. In N. K. Denzin (Ed.), Sociological Methods (1st ed., pp. 105–114). Routledge.

[R47] Glaser B. G., & Strauss A. L. (2014). Applying grounded theory. The Grounded Theory Review (Vol. 13, pp. 46–50). Sociology Press.

[R48] Glaser B., & Strauss A. (1967). The Discovery of Grounded Theory: Strategies for Qualitative Research. Sociology Press.

[R49] Glaser B. G. Strauss A. L. Strutzel E. (1968). The discovery of grounded theory; strategies for qualitative research. Nurs Res, 17, 364.

[R50] Glaser B. G., & Holton J. (2004). Remodeling grounded theory. Forum, Qual Soc Res, 5, 1–22.

[R51] Gomaa M. A. M. Elmagd M. H. A. Elbadry M. M. Kader R. M. A. (2014). Depression, Anxiety and Stress Scale in patients with tinnitus and hearing loss. Eur Arch Otorhinolaryngol, 271, 2177–2184. 24071860 10.1007/s00405-013-2715-6

[R52] Hall W. A., & Callery P. (2001). Enhancing the rigor of grounded theory: Incorporating reflexivity and relationality. Qual Health Res, 11, 257–272. 11221119 10.1177/104973201129119082

[R53] Hallam R. Ashton P. Sherbourne K. Gailey L. (2006). Acquired profound hearing loss: Mental health and other characteristics of a large sample. Int J Audiol, 45, 715–723. 17132560 10.1080/14992020600957335

[R54] Hallam R. Ashton P. Sherbourne K. Gailey L. (2008). Persons with acquired profound hearing loss (APHL): How do they and their families adapt to the challenge? Health (London), 12, 369–388. 18579633 10.1177/1363459308090054

[R55] Hallberg L. R. (1999). Hearing impairment, coping, and consequences on family life. J Acad Rehabil Audiol, 32, 45–59.

[R56] Hallberg L. R. (2006). The “core category” of grounded theory: Making constant comparisons. Int J Qual Stud Health Well-being, 1, 141–148.

[R57] Heffernan E. Coulson N. S. Henshaw H. Barry J. G. Ferguson M. A. (2016). Understanding the psychosocial experiences of adults with mild-moderate hearing loss: An application of Leventhal’s self-regulatory model. Int J Audiol, 55, S3–S12. 10.3109/14992027.2015.1117663 PMC5706634 26754550

[R58] Hricová M. (2018). Coping strategies and social environment of patients with sudden hearing loss. Health Psychology Report, 6, 216–221.

[R59] Hughes P. A. (2001). Self-concept of hard of hearing young adults: a grounded theory. Retrieved from (Doctoral dissertation). Retrieved from ProQuest Dissertations and Theses database. (Accession Order No. 304741189).

[R60] Hughes S. E. Hutchings H. A. Rapport F. L. McMahon C. M., & Boisvert I. (2018). Social Connectedness and Perceived Listening Effort in Adult Cochlear Implant Users: A Grounded Theory to Establish Content Validity for a New Patient-Reported Outcome Measure. Ear and hearing, 39, 922–934. 10.1097/AUD.0000000000000553 29424766

[R61] Hussein M. E. Hirst S. Salyers V. Osuji J. (2014). Using grounded theory as a method of inquiry: Advantages and disadvantages. Qual Rep, 19, 1–15.

[R62] Hutchison A. J. Johnston L. Breckon J. (2011). Grounded theory-based research within exercise psychology: A critical review. Qual Res Psychol, 8, 247–272.

[R63] Jennings M. B., & Shaw L. (2008). Impact of hearing loss in the workplace: Raising questions about partnerships with professionals. Work, 30, 289–295. 18525152

[R64] Kenny M., & Fourie R. (2015). Contrasting classic, Straussian, and constructivist grounded theory: Methodological and philosophical conflicts. Qual Rep, 20, 1270–1289.

[R65] Knudsen L. V. Laplante-Lévesque A. Jones L. Preminger J. E. Nielsen C. Lunner T. Hickson L. Naylor G. Kramer S. E. (2012). Conducting qualitative research in audiology: A tutorial. Int J Audiol, 51, 83–92. 21916797 10.3109/14992027.2011.606283

[R66] Koerber R. M. Moodie S. Jennings M. B. (2022). The experiences of telepractice nurses undertaking a vocational audiological rehabilitation program. Int J Audiol, 61, 390–399. 34319816 10.1080/14992027.2021.1951854

[R67] Lazenbatt A., & Elliott N. (2005). How to recognise a ‘quality’ grounded theory research study. Aust J Adv Nurs, 22, 48. 16499241

[R68] Lomborg K., & Kirkevold M. (2003). Truth and validity in grounded theory—A reconsidered realist interpretation of the criteria: Fit, work, relevance and modifiability. Nurs Philos, 4, 189–200. 12969449 10.1046/j.1466-769x.2003.00139.x

[R69] Martin D. M. (2010). Learning to listen: the voices of post-secondary deaf and hard of hearing learners. (Doctoral dissertation, University of Alberta, Canada). ProQuest Dissertations and Theses, 199-n/a.

[R70] McCann T., & Polacsek M. (2018). Understanding, choosing and applying grounded theory: Part 1. Nurse Res, 26, 36–41. 10.7748/nr.2018.e1592 30488674

[R71] McCrae N., & Purssell E. (2016). Is it really theoretical? A review of sampling in grounded theory studies in nursing journals. J Adv Nurs, 72, 2284–2293. 27113800 10.1111/jan.12986

[R72] McRackan T. R. Velozo C. A. Holcomb M. A. Camposeo E. L. Hatch J. L. Meyer T. A. Lambert P. R. Melvin C. L., & Dubno J. R. (2017). Use of adult patient focus groups to develop the initial item bank for a cochlear implant quality-of-life instrument. JAMA Otolaryngol Head Neck Surg, 143, 975–982. 28772297 10.1001/jamaoto.2017.1182 PMC5710256

[R73] Meston C., & Ng S. (2012). A grounded theory primer for audiology. In Seminars in Hearing (Vol. 33, No. 2, pp. 135–146). Thieme Medical Publishers. 10.1055/s-0032-1311674

[R74] Mick P. Kawachi I. Lin F. R. (2014). The association between hearing loss and social isolation in older adults. Otolaryngol Head Neck Surg, 150, 378–384. 24384545 10.1177/0194599813518021

[R75] Miller A. (1995). Building grounded theory within educational psychology practice. Educ Child Psychol, 12, 5–14.

[R76] Moher D. Clarke M. Ghersi D. Liberati A. Petticrew M. Shekelle P. G. Stewart L. (2015). Referred reporting items for systematic review and meta-analysis protocols (PRISMA-P) 2015 statement. Syst Rev, 4, 1–9. 25554246 10.1186/2046-4053-4-1 PMC4320440

[R77] Morse J. M., & Clark L. (2019). The nuances of grounded theory sampling and the pivotal role of theoretical sampling. In A. Bryant, & K. Charmaz (Eds.), The SAGE Handbook of Current Developments in Grounded Theory (pp. 145–166). SAGE Publications Ltd. 10.4135/9781526485656

[R78] Morse J. M. Stern P. N. Corbin J. Bowers B. Charmaz K., & Clarke A. E. (2016). Developing Grounded Theory: The Second Generation. Routledge.

[R79] Nordvik O. Laugen Heggdal P. O. Brännström J. Vassbotn F. Aarstad A. K. Aarstad H. J. (2018). Generic quality of life in persons with hearing loss: A systematic literature review. BMC Ear Nose Throat Disord, 18, 11. 29386982 10.1186/s12901-018-0051-6 PMC5778781

[R80] Nova F. F. Rifat M. R. Saha P. Ahmed S. I. Guha S. (2018). Silenced voices: Understanding sexual harassment on anonymous social media among Bangladeshi people. Companion of the 2018 ACM Conference on Computer Supported Cooperative Work and Social Computing, (CSCW’18), 209212.

[R81] Otake Y. (2019). Suffering of silenced people in northern Rwanda. Soc Sci Med, 222, 171–179. 30658290 10.1016/j.socscimed.2019.01.005

[R82] Pryce H. Hall A. Laplante-Lévesque A. Clark E. (2016). A qualitative investigation of decision making during help-seeking for adult hearing loss. Int J Audiol, 55, 658–665. 27385528 10.1080/14992027.2016.1202455

[R83] Punch J. L. Hitt R. Smith S. W. (2019). Hearing loss and quality of life. J Commun Disord, 78, 33–45. 30639959 10.1016/j.jcomdis.2019.01.001

[R84] RNID. (2023). Facts and figures, 2020. Action on Hearing Loss. Retrieved November 14, 2023 from https://rnid.org.uk/get-involved/research-and-policy/facts-and-figures/.

[R85] Rolfe G. (2006). Validity, trustworthiness and rigour: Quality and the idea of qualitative research. J Adv Nurs, 53, 304–310. 16441535 10.1111/j.1365-2648.2006.03727.x

[R86] Sbaraini A. Carter S. M. Evans R. Blinkhorn A. (2011). How to do a grounded theory study: A worked example of a study of dental practices. BMC Med Res Methodol, 11, 128–128. 21902844 10.1186/1471-2288-11-128 PMC3184112

[R87] Scinicariello F. Carroll Y. Eichwald J. Decker J. Breysse P. N. (2019). Association of obesity with hearing impairment in adolescents. Sci Rep, 9, 1877. 30755633 10.1038/s41598-018-37739-5 PMC6372622

[R88] Shaw L. Tetlaff B. Jennings M. B. Southall K. E. (2013). The standpoint of persons with hearing loss on work disparities and workplace accommodations. Work, 46, 193–204. 24004807 10.3233/WOR-131741

[R89] Smith K., & Biley F. (1997). Understanding grounded theory: Principles and evaluation. Nurse Res, 4, 17–30. 27285771 10.7748/nr.4.3.17.s3

[R90] Stol K.-J. Ralph P. Fitzgerald B. (2016). Grounded theory in software engineering research: A critical review and guidelines. Proceedings of the 38th International Conference on Software Engineering (ICSE) (Vol. 14–22, pp. 120–131.

[R91] Strauss A. L., & Corbin J. M. (1997). Grounded Theory in Practice. Sage Publications Inc.

[R92] Svinndal E. V. Jensen C. Rise M. B. (2020). Working life trajectories with hearing impairment. Disabil Rehabil, 42, 190–200. 30298745 10.1080/09638288.2018.1495273

[R93] Thomson S. B. (2010). Grounded theory-sample size. J Adm Gov, 5, 45–52.

[R94] Thornberg R. (2012). Informed grounded theory. Scand J Educ Res, 56, 243–259.

[R95] Thornberg R. Perhamus L., & Charmaz K. (2014). Grounded theory. In Saracho O. N. (Ed.). Handbook of Research Methods in Early Childhood Education: Research Methodologies (Vol. 1, pp. 405–439). Information Age Publishing.

[R96] Timonen V. Foley G. Conlon C. (2018). Challenges when using grounded theory: A pragmatic introduction to doing GT research. Int J Qual Methods, 17, 1609406918758086.

[R97] Urquhart C. Lehmann H. Myers M. D. (2010). Putting the ‘theory’ back into grounded theory: Guidelines for grounded theory studies in information systems. Inf Syst J, 20, 357–381.

[R98] Vos T. Allen C. Arora M. Barber R. M. Bhutta Z. A. Brown A. Carter A. Casey D. C. Charlson F. J. Chen A. Z. (2016). Global, regional, and national incidence, prevalence, and years lived with disability for 310 diseases and injuries, 1990–2015: A systematic analysis for the Global Burden of Disease Study 2015. Lancet, 388, 1545–1602. 27733282 10.1016/S0140-6736(16)31678-6 PMC5055577

[R99] Ward K. Hoare K. J. Gott M. (2015). Evolving from a positivist to constructionist epistemology while using grounded theory: Reflections of a novice researcher. J Res Nurs, 20, 449–462.

[R100] Warringa L. T. Henke C. E. Pronk M. Kramer S. E. Stam M. (2020). Relationships between coping behaviors and social loneliness in adults with self-reported hearing problems. Ear Hear, 41, 1040–1050. 31977728 10.1097/AUD.0000000000000828

[R101] Weed M. (2009). Research quality considerations for grounded theory research in sport & exercise psychology. Psychol Sport Exerc, 10, 502–510.

[R102] World Health Organization, & Public Health Agency of Canada. (2005). Preventing chronic diseases: a vital investment. World Health Organization.

[R103] World Health Organization. 2018. Addressing the rising prevalence of hearing loss. World Health Organization. https://apps.who.int/iris/handle/10665/260336.

