# Supplementary material for: Applications of Grounded Theory Methodology to Investigate Hearing Loss: A Methodological Qualitative Systematic Review With Developed Guidelines
Source: Ear Hear. 2024 Apr 14;45(3):550–62. doi: 10.1097/AUD.0000000000001459 (PMC11008453; doi:10.1097/AUD.0000000000001459)
Supplement: Supplementary file 1 [file aud-45-550-s001.pdf]

Supplemental Digital Content 1

Table 1: The schools of grounded theory methodology

**Dimensions of Comparison**	**Classic/ Glaserian School (1967)**	**Straussain School (1990)**	**Constructivist School (2006)**
**School founders**	Barney Glaser & Anselm Strauss	Anselm Strauss & Juliet Corbin	Kathy Charmaz
**Philosophical stance**	Empiricism & Soft Positivism	Symbolic Interactionism & Interpretivism	Social Constructivism & Symbolic Interactionism
**Aim**	Observe patterns of behaviour within a social context & discover a grounded theory	Explain social phenomena & create a grounded theory	Examine actions and social processes & construct a grounded theory
**Philosophical principle**	Knowledge can be objectively measured and is formed based on an individual's experience	Knowledge is subjective and socially constructed. Establishing one truth is impossible	Knowledge is subjective and is constructed by both the participant's experiences and the researcher's own understandings
**Analysis and Coding Techniques**	Open, selective, and theoretical coding	Open, selective, and axial coding (theory modelling) with matrix building	Initial, focused, and theoretical coding
**Researcher's influence**	Passive: Researchers can completely detach themselves from their research and have no influence	Active: Researchers cannot detach themselves from their research and will always influence the research process and findings	Active: A comprehensive truth is pursued; however, it will only be reflective of the social context and group studied

**Literature Review**	Perform literature review only after data has been collected and analysed	Preliminary literature review can be formed before data collection to enhance theoretical sensitivity. More in-depth review conducted after data analysis	Preliminary literature review can be formed before data collection to enhance theoretical sensitivity. More in-depth review conducted after data analysis
**Grounded theory emphasis**	*Constant comparative analysis*: constantly comparing data and outcomes to establish objective knowledge	*Reflexivity*: researcher provides reflections on the process of data collection, analysis, and recognises how they influence this process	*Constant comparative analysis, reflexivity, theoretical sampling* (knowledge is pursued and collected through recruiting different samples and investigating different concepts to develop the theory)
