# Supplementary material for: Applications of Grounded Theory Methodology to Investigate Hearing Loss: A Methodological Qualitative Systematic Review With Developed Guidelines
Source: Ear Hear. 2024 Apr 14;45(3):550–62. doi: 10.1097/AUD.0000000000001459 (PMC11008453; doi:10.1097/AUD.0000000000001459)
Supplement: Supplementary file 2 [file aud-45-550-s002.pdf]

Supplemental Digital Content 2: GUREGT TOOL

Main area	IItem	Grounded theory methodology		
		Glaser	Strauss and Corbin	Charmaz
**Study aim**	1	Is the grounded theory study aim presented to generate a theory of patterns of behaviour?	Is the grounded theory study aim presented to develop a well-integrated set of concepts that provide a theoretical explanation of a social phenomenon?	Is the grounded theory study aim presented to construct a theory focusing on examining process and actions?
**Philosophical framework**	2	Is the grounded theory embedded in any philosophical background? Why and how?	How is the grounded theory embedded in symbolic interactionism?	Is the grounded theory embedded in symbolic interactionism and social constructivism?
**The researchers' role**	3	Is the researcher's theoretical sensitivity described according to conceptual thinking, level of insight into the research area and ability to generate meaning from data?	Is the researchers' theoretical sensitivity according to theoretical insight, professional and personal experience, described and explained?	Is the researchers' reflective and interpretive stance in a two-way interaction with the participant described and explained?
**Data collection**	4	Is data collection methods described and explained?	Is data collection methods described and explained?	Is data collection methods described and explained?
	5	Has qualitative or quantitative data collection methods been used? How and why?	Has qualitative or quantitative data collection methods been used? How and why?	Has qualitative or quantitative data collection methods been used? How and why?
**Memos**	6	Has memos been written throughout the study about concepts and categories and are they used to formulate and generate the theory?	Has field notes and diagramming been written and used throughout the study about concepts and categories and are they used to formulate and develop the theory?	Has memos been written throughout the study about concepts and categories and are they used to formulate and construct the theory?
**Sampling procedures**	7	Is initial sampling conducted in the beginning of data collection described and explained?	Is open, relational and variational sampling conducted in the beginning of data collection described and explained?	Is initial sampling conducted in the beginning of data collection described and explained?
	8	Is theoretical sampling of the emerging categories and theory from the data collection described and explained?	Is theoretical and discriminate sampling of the emerging categories and theory from the data collection described and explained?	Is theoretical sampling of the emerging categories and theory from the data collection described and explained?
	9	Is the selection of participants guided by theoretical sampling? How?	Is the selection of participants guided by theoretical sampling? How?	Is the selection of participants guided by theoretical sampling? How?
**Theoretical saturation**	10	Is the reach of theoretical saturation explained according to no new insights relevant for the emergent theory?	Is the reach of theoretical saturation explained according to no new insights relevant for the emergent theory?	Is the reach of theoretical saturation explained according to no new insights relevant for the concepts and categories and the emergent theory?
**Analysis and coding**	11	Is the coding levels and concurrent process of coding described according to open, selective and theoretical coding?	Is the coding levels and concurrent process of coding described according to open, axial (the paradigm model) and selective coding? And is matrix building and storyline applied and described? How?	Is the coding levels and concurrent process of coding described according to initial, focused and theoretical coding?
	12	Which concepts has guided the specific coding levels and how?	Which categories has guided the specific coding levels and how?	Which codes has guided the specific coding levels and how?
	13	Is the core category identified before conducting selective coding?	Is the central category identified before conducting selective coding?	Is the basic social process identified before conducting focused coding?
	14	Which theoretical codes have structured the theory to a progressive level of abstraction?	Which categories have contributed to identify the density, internal consistency and gaps in logic of the parsimonious theory?	Which theoretical codes have structured the theory to a progressive level of abstraction?
	15	Is the constant comparison method used to compare incidents with incidents, incidents with categories and categories with categories?	Is the constant comparison method used to compare incidents with incidents, incidents with categories and categories with categories?	Is the constant comparison method used to compare incidents with incidents, incidents with categories and categories with categories?
	16	Is the simultaneous data collection, analysis and coding guided by the theoretical sampling and writing memos described and explained?	Is the simultaneous data collection, analysis and coding guided by the theoretical sampling and writing memos described and explained?	Is the simultaneous data collection, analysis and coding guided by the theoretical sampling and writing memos described and explained?

	IItem	Grounded theory methodology		
		**Glaser**	**Strauss and Corbin**	**Charmaz**
Review of literature	17	Is the literature reviewed avoided initially in the grounded theory study? Why and how?	Is general literature reviewed initially in the grounded theory study to assist in formulating questions? Why and how?	Is the literature reviewed initially in the grounded theory study to expand the contextual framework? Why and how?
	18	Is the literature reviewed after theory development on the basis of the emerging concepts and theory? How and on what grounds?	Is an extensive literature reviewed performed after theory development on the basis of the emerging concepts and theory? How and on what grounds?	Is the literature reviewed during theory development on the basis of the emerging concepts and theory? How and on what grounds?
Results/ the theory	19	Is the main concern presented and explained?	Is the main concern presented and explained?	Is the main social interactions of the theory presented and explained
	20	Is the core category and the related categories presented and explained?	Are the central category and the related categories, properties and dimensions presented and described?	Is the basic social process and the related categories presented and explained?
	21	Does the theory account for the overall pattern of behaviour in the substantive area?	Does the theory provide a thorough theoretical explanation of the social phenomenon?	Does the theory account for the essential processes and actions in the social interactions of the participants?
	22	Is conceptualization used rather than description using quotes when writing the theory?	Are quotes used and argued for to describe the theory?	Are quotes used and argued for to describe the theory?
Discussion	23	Are the key relationships between the core category and concepts discussed and related to relevant literature?	Are the key relationships between the central category and categories discussed and related to relevant literature?	Are the key relationships between the categories and codes discussed and related to relevant literature?
Evaluation criteria	24	Are the criteria of fit, work, relevance, and modifiability presented and explained?	Are the criteria of validity, reliability and credibility of data presented and explained?	Are the criteria of credibility, originality, resonance, and usefulness, as well as fit, work, relevance, and modifiability presented and explained?
	25	Are the evaluation criteria used to evaluate the theory?	Are the evaluation criteria used to evaluate the theory?	Are the evaluation criteria used to evaluate the theory?
