# Supplementary material for: Applications of Grounded Theory Methodology to Investigate Hearing Loss: A Methodological Qualitative Systematic Review With Developed Guidelines
Source: Ear Hear. 2024 Apr 14;45(3):550–62. doi: 10.1097/AUD.0000000000001459 (PMC11008453; doi:10.1097/AUD.0000000000001459)
Supplement: Supplementary file 3 [file aud-45-550-s003.pdf]

Supplemental Digital Content 3: Supplemental Search Strategy

Search Database	Intial search Results	Updated search results (March 2020)	Updated search results (August 2021)	Total
Applied Social Sciences Index and Abstracts (1987–current)	8	11	0	19
British Nursing index (1994–current)	8	11	0	19
Cumulative Index to Nursing and Allied Health Literature (1961–current)	56	4	0	60
Global Health (OvidSP database, 1973–current)	0	0	0	0
MEDLINE (Ovid, In-Process & Other Non-Indexed Citations, 1946–current)	40	6	13	59
PsycINFO (1800s–current)	49	4	10	63
PubMed (1996–current)	98	6	24	128
Scopus (1983–current)	57	9	9	75
Web of Science (1899–current)	56	12	8	76
**Intial Total**	**372**	**63**	**64**	**499**
**Duplicates**	**249**	**47**	**48**	**344**
**Total After duplication removal**	**123**	**16**	**16**	**155**

Applied Social Sciences Index and Abstracts (1987–current): 19

(((((((((((((((((((MAINSUBJECT.EXACT.EXPLODE("Hearing impaired people") OR MAINSUBJECT.EXACT.EXPLODE("Communication Profile for the Hearing Impaired")) OR su(communication partner*) OR su(audiologist*) OR MAINSUBJECT.EXACT.EXPLODE("Audiology")) AND stype.exact("Scholarly Journals")) AND la.exact("English")) AND stype.exact("Scholarly Journals")) AND la.exact("English")) AND stype.exact("Scholarly Journals")) AND la.exact("English")) AND stype.exact("Scholarly Journals")) AND la.exact("English")) AND stype.exact("Scholarly Journals")) AND la.exact("English")) AND stype.exact("Scholarly Journals")) AND la.exact("English")) AND stype.exact("Scholarly Journals")) AND la.exact("English")) AND PEER(yes)) OR ((((((((((((((((MAINSUBJECT.EXACT.EXPLODE("Hearing aids") OR MAINSUBJECT.EXACT.EXPLODE("Cochlear implants")) AND stype.exact("Scholarly Journals")) AND la.exact("English")) AND stype.exact("Scholarly Journals")) AND la.exact("English")) AND stype.exact("Scholarly Journals")) AND la.exact("English")) AND stype.exact("Scholarly Journals")) AND la.exact("English")) AND stype.exact("Scholarly Journals")) AND la.exact("English")) AND stype.exact("Scholarly Journals")) AND la.exact("English")) AND stype.exact("Scholarly Journals")) AND la.exact("English")) AND PEER(yes)) OR ((((((((((((((((su(hearing loss*) OR (MAINSUBJECT.EXACT.EXPLODE("Hearing impairment") OR MAINSUBJECT.EXACT("Noise induced hearing impairment") OR MAINSUBJECT.EXACT("Occupational hearing impairment") OR MAINSUBJECT.EXACT("Sensorineural hearing impairment")) OR MAINSUBJECT.EXACT("Sudden hearing loss") OR su(hearing disorder*) OR su(auditory impairment*) OR su(loss of hearing*)) AND stype.exact("Scholarly Journals")) AND la.exact("English")) AND stype.exact("Scholarly Journals")) AND la.exact("English")) AND stype.exact("Scholarly Journals")) AND la.exact("English")) AND stype.exact("Scholarly Journals")) AND la.exact("English")) AND stype.exact("Scholarly Journals")) AND la.exact("English")) AND stype.exact("Scholarly Journals")) AND la.exact("English")) AND stype.exact("Scholarly Journals")) AND la.exact("English")) AND PEER(yes))) AND (MAINSUBJECT.EXACT.EXPLODE("Grounded theory") AND PEER(yes))) AND (stype.exact("Scholarly Journals") AND PEER(yes))

British Nursing index: 19

(((((((((((((((((((MAINSUBJECT.EXACT.EXPLODE("Hearing impaired people") OR MAINSUBJECT.EXACT.EXPLODE("Communication Profile for the Hearing Impaired")) OR su(communication partner*) OR su(audiologist*) OR MAINSUBJECT.EXACT.EXPLODE("Audiology")) AND stype.exact("Scholarly Journals")) AND la.exact("English")) AND stype.exact("Scholarly Journals")) AND la.exact("English")) AND stype.exact("Scholarly Journals")) AND la.exact("English")) AND stype.exact("Scholarly Journals")) AND la.exact("English")) AND stype.exact("Scholarly

Journals")) AND la.exact("English")) AND stype.exact("Scholarly Journals")) AND la.exact("English")) AND stype.exact("Scholarly Journals")) AND la.exact("English")) AND PEER(yes)) OR (((((((((((((((MAINSUBJECT.EXACT.EXPLODE("Hearing aids") OR MAINSUBJECT.EXACT.EXPLODE("Cochlear implants")) AND stype.exact("Scholarly Journals")) AND la.exact("English")) AND stype.exact("Scholarly Journals")) AND la.exact("English")) AND stype.exact("Scholarly Journals")) AND la.exact("English")) AND stype.exact("Scholarly Journals")) AND la.exact("English")) AND stype.exact("Scholarly Journals")) AND la.exact("English")) AND stype.exact("Scholarly Journals")) AND la.exact("English")) AND stype.exact("Scholarly Journals")) AND la.exact("English")) AND PEER(yes)) OR (((((((((((((((su(hearing loss*) OR (MAINSUBJECT.EXACT.EXPLODE("Hearing impairment") OR MAINSUBJECT.EXACT("Noise induced hearing impairment") OR MAINSUBJECT.EXACT("Occupational hearing impairment") OR MAINSUBJECT.EXACT("Sensorineural hearing impairment")) OR MAINSUBJECT.EXACT("Sudden hearing loss") OR su(hearing disorder*) OR su(auditory impairment*) OR su(loss of hearing*)) AND stype.exact("Scholarly Journals")) AND la.exact("English")) AND stype.exact("Scholarly Journals")) AND la.exact("English")) AND stype.exact("Scholarly Journals")) AND la.exact("English")) AND stype.exact("Scholarly Journals")) AND la.exact("English")) AND stype.exact("Scholarly Journals")) AND la.exact("English")) AND stype.exact("Scholarly Journals")) AND la.exact("English")) AND stype.exact("Scholarly Journals")) AND la.exact("English")) AND PEER(yes))) AND (MAINSUBJECT.EXACT.EXPLODE("Grounded theory") AND PEER(yes))) AND (stype.exact("Scholarly Journals") AND PEER(yes))

Cumulative Index to Nursing and Allied Health Literature- EBSCOhost: 60

((MH "Hearing Loss, Functional") OR (MM "Rehabilitation of Hearing Impaired") OR (MH "Hearing Loss, Partial") OR (MH "Hearing Screening") OR (MH "Hearing Loss, Conductive") OR (MH "Hearing Loss, High-Frequency") OR (MH "Hearing Loss, Noise-Induced") OR (MH "Hearing Loss, Sensorineural") OR "hearing impairment*" OR (MM "Hearing Loss, Central") OR (MM "Hearing Disorders") OR (MH "Hearing Aid Fitting") OR (MM "Hearing Aids") OR (MH "Hearing Aid Care (Saba CCC)") (MH "Hearing Loss, Sensorineural") OR (MH "Hearing Loss, Noise-Induced") OR (MH "Hearing Loss, High-Frequency") OR (MH "Hearing Loss, Conductive") OR (MH "Presbycusis") OR "hearing loss*" (MH "Hearing Aid Fitting") OR (MH "Hearing Aid Care (Saba CCC)") OR (MH "Hearing Aid Care") OR (MM "Hearing Aids+") (MH "Cochlear Implant+") OR (MH "Cochlear Implant Programming") "hard of hearing" OR "people with hearing loss*" OR "Person with hearing loss*" OR "hearing impaired*" OR (MH "Audiologist Attitudes") OR (MM "Audiologists") OR "Audiologist*" OR "Communication Partner") AND (MM "Grounded Theory") OR "grounded theory*".))

Global Health: 0

1. Hearing Loss, Central/ or Hearing Loss, Conductive/ or Hearing Loss, Sensorineural/ or Hearing Loss, Sudden/ or Hearing Loss, Functional/ or Hearing Loss, High-Frequency/ or Hearing Loss, Noise-Induced/ or Hearing Disorders/ or Hearing Loss, Bilateral/ or Hearing Loss, Unilateral/ or Hearing Loss, Mixed Conductive-Sensorineural/ or Hearing Loss/
2. exp Hearing Loss/ or hearing loss*.mp.
3. 1 or 2
4. Amplifiers, Electronic/ or amplifier*.mp.
5. listening device*.mp. or Hearing Aids/
6. Cochlear Implants/ or Cochlear Implantation/ or cochlear implant*.mp.
7. exp Hearing Aids/ or hearing aid*.mp.
8. 4 or 5 or 6 or 7
9. hard of hearing*.mp.
10. communication partner*.mp.
11. audiologist*.mp. or Audiologists/ or Audiology/
12. exp Persons With Hearing Impairments/
13. 9 or 10 or 11 or 12
14. exp Grounded Theory/
15. grounded theory*.mp.
16. 14 or 15
17. 3 or 8 or 13
18. 16 and 17

Medline Ovid: 59

1. Hearing Loss, Central/ or Hearing Loss, Conductive/ or Hearing Loss, Sensorineural/ or Hearing Loss, Sudden/ or Hearing Loss, Functional/ or Hearing Loss, High-Frequency/ or Hearing Loss, Noise-Induced/ or Hearing Disorders/ or Hearing Loss, Bilateral/ or Hearing Loss, Unilateral/ or Hearing Loss, Mixed Conductive-Sensorineural/ or Hearing Loss/
2. exp Hearing Loss/ or hearing loss*.mp.
3. 1 or 2
4. Amplifiers, Electronic/ or amplifier*.mp.
5. listening device*.mp. or Hearing Aids/
6. Cochlear Implants/ or Cochlear Implantation/ or cochlear implant*.mp.
7. exp Hearing Aids/ or hearing aid*.mp.
8. 4 or 5 or 6 or 7
9. hard of hearing*.mp.
10. communication partner*.mp.

11. audiologist*.mp. or Audiologists/ or Audiology/
12. exp Persons With Hearing Impairments/
13. 9 or 10 or 11 or 12
14. exp Grounded Theory/
15. grounded theory*.mp.
16. 14 or 15
17. 3 or 8 or 13
18. 16 and 17

PsycINFO: 63

(((((((((((((((((((MAINSUBJECT.EXACT.EXPLODE("Hearing impaired people") OR MAINSUBJECT.EXACT.EXPLODE("Communication Profile for the Hearing Impaired")) OR su(communication partner*) OR su(audiologist*) OR MAINSUBJECT.EXACT.EXPLODE("Audiology")) AND stype.exact("Scholarly Journals")) AND la.exact("English")) AND stype.exact("Scholarly Journals")) AND la.exact("English")) AND stype.exact("Scholarly Journals")) AND la.exact("English")) AND stype.exact("Scholarly Journals")) AND la.exact("English")) AND stype.exact("Scholarly Journals")) AND la.exact("English")) AND stype.exact("Scholarly Journals")) AND la.exact("English")) AND stype.exact("Scholarly Journals")) AND la.exact("English")) AND PEER(yes)) OR ((((((((((((((((MAINSUBJECT.EXACT.EXPLODE("Hearing aids") OR MAINSUBJECT.EXACT.EXPLODE("Cochlear implants")) AND stype.exact("Scholarly Journals")) AND la.exact("English")) AND stype.exact("Scholarly Journals")) AND la.exact("English")) AND stype.exact("Scholarly Journals")) AND la.exact("English")) AND stype.exact("Scholarly Journals")) AND la.exact("English")) AND stype.exact("Scholarly Journals")) AND la.exact("English")) AND stype.exact("Scholarly Journals")) AND la.exact("English")) AND stype.exact("Scholarly Journals")) AND la.exact("English")) AND PEER(yes)) OR ((((((((((((((((su(hearing loss*) OR (MAINSUBJECT.EXACT.EXPLODE("Hearing impairment") OR MAINSUBJECT.EXACT("Noise induced hearing impairment") OR MAINSUBJECT.EXACT("Occupational hearing impairment") OR MAINSUBJECT.EXACT("Sensorineural hearing impairment")) OR MAINSUBJECT.EXACT("Sudden hearing loss") OR su(hearing disorder*) OR su(auditory impairment*) OR su(loss of hearing*)) AND stype.exact("Scholarly Journals")) AND la.exact("English")) AND stype.exact("Scholarly Journals")) AND la.exact("English")) AND stype.exact("Scholarly Journals")) AND la.exact("English")) AND stype.exact("Scholarly Journals")) AND la.exact("English")) AND stype.exact("Scholarly Journals")) AND la.exact("English")) AND stype.exact("Scholarly Journals")) AND la.exact("English")) AND stype.exact("Scholarly Journals")) AND la.exact("English")) AND PEER(yes))) AND

(MAINSUBJECT.EXACT.EXPLODE("Grounded theory") AND PEER(yes))) AND (stype.exact("Scholarly Journals") AND PEER(yes))

PubMed: 128

(grounded theory*) AND (((((((hard of hearing*.mp.[MeSH Terms]) OR communication partner*.mp.[MeSH Terms]) OR (audiologist*.mp. OR Audiologists/ OR Audiology/[MeSH Terms])) OR exp Persons With Hearing Impairments/[MeSH Terms])) OR (((((Amplifiers, Electronic/ OR amplifier*.mp.[MeSH Terms])) OR (Hearing Aids/ OR listening device*.mp.[MeSH Terms])) OR (Cochlear Implants/ OR cochlear implant*.mp. OR Cochlear Implantation/[MeSH Terms])) OR (exp Hearing Aids/ OR hearing aid*.mp.[MeSH Terms]))) OR (((hearing impairment*[MeSH Terms]) OR (Hearing Loss, Noise-Induced/ OR Hearing Loss/ OR Hearing Loss, Sensorineural/ OR Hearing Loss, Unilateral/ OR Hearing Disorders/ OR Hearing Loss, Sudden/ OR Hearing Loss, Bilateral/ OR Hearing Tests/ OR Hearing Loss, Conductive/[MeSH Terms])) OR (hearing loss*.mp. OR exp Hearing Loss/[MeSH Terms])))

Scopus: 75

((TITLE-ABS-KEY (hearing AND impairment*) OR TITLE-ABS-KEY (hearing AND loss*) OR TITLE-ABS-KEY (auditory AND impairment*) OR TITLE-ABS-KEY (hearing AND disorder*) OR TITLE-ABS-KEY (loss AND of AND hearing*)) OR (TITLE-ABS-KEY (hearing AND aids*) OR TITLE-ABS-KEY (listening AND device*) OR TITLE-ABS-KEY (cochlear AND implant*)) OR (TITLE-ABS-KEY (hard AND of AND hearing*) OR TITLE-ABS-KEY (people AND with AND hearing AND loss*) OR TITLE-ABS-KEY (person AND with AND hearing AND loss*) TITLE-ABS-KEY (hearing AND impaired*) OR TITLE-ABS-KEY (audiologist*) OR TITLE-ABS-KEY (communication AND partner*))) AND (TITLE-ABS-KEY (grounded AND theory*)) AND (LIMIT-TO (LANGUAGE , "English")) AND (LIMIT-TO (DOCTYPE , "ar"))

Web of Science: 76

(TS=(Hearing impairment* or hearing loss* or auditory impairment* or hearing disorder*)) *AND* LANGUAGE: (English) *AND* DOCUMENT TYPES: (Article), OR (TS=(Hearing aid* or listening device* or cochlear
implant*)) OR *AND* LANGUAGE: (English) *AND* DOCUMENT TYPES: (Article)), OR (TS=(hard of hearing* or person with hearing loss* or people with hearing loss* or audiologist* or communication partner* hearing impaired
people*) OR *AND* LANGUAGE: (English) *AND* DOCUMENT TYPES: (Article) AND TS=(Grounded
theory*)) *AND* LANGUAGE: (English) *AND* DOCUMENT TYPES: (Article))
