# Supplementary material for: Applications of Grounded Theory Methodology to Investigate Hearing Loss: A Methodological Qualitative Systematic Review With Developed Guidelines
Source: Ear Hear. 2024 Apr 14;45(3):550–62. doi: 10.1097/AUD.0000000000001459 (PMC11008453; doi:10.1097/AUD.0000000000001459)
Supplement: Supplementary file 5 [file aud-45-550-s005.pdf]

Supplemental Digital Content 5 - GUREGT tool critical appraisal of included studies

Table 2. Included studies and scored grounded theory applications

**Author(s) & Publication year**	**Area of Hearing loss Research**	**Aims**	**Sample**	**Methods**	**School of Grounded theory followed**	**Main GT Principles not met**	**Main GT Principles met**	**Theory Developed**	**Overall GUREGT score (out of 50)**
Almotiri, 2017	Living with Hearing loss: Adaptations to life transitions	Explore the transitioning process of Deaf/ hard of hearing Saudi students in a foreign (US) university	N=5 Saudi national PHL students with studying at university in the US.	Demographic questionnaires and semi-structured interviews	Constructivist school (stated)	No theoretical sampling, no constant comparative analysis (CCA), no simultaneous data collection & analysis	Reflexivity, memoing, initial & focused coding	No	19
Adler, 2018	Identity and hearing loss	Examine hearing loss identity, and identify how it impacts personality development	N=1 Female with severe to profound HL	Semi-structured interview & personality questionnaires	Constructivist school (implied)	No theoretical sampling, no initial coding, no CCA, no simultaneous data collection & analysis or memoing	Focused coding, Reflections, member checking, focused coding	No	10
Bell, Carl, & Swart, 2016	Identity and hearing loss	Assess how self-identity influences PHL's choice to disclose disability	N=7 Students with hearing loss studying at University of Stellenbosch, South Africa.	In-depth interviews & demographic questionnaire	Constructivist school (stated)	No theoretical sampling, no CCA, no simultaneous data collection & analysis	Memoing, crystallization, theoretical coding, focused coding	No	25

Carson, 2005	Help-seeking behaviours for hearing loss	Identify help-seeking for age-related hearing loss in PHL with presbycusis	N=14 Female PHL with presbycusis n=7; family members n=6; audiologist n=1.	Participant observation & semi-structured interviews	Pragmatic school (implied)	No theoretical sampling, no reflexivity, no simultaneous data collection & analysis	Open and selective coding, memoing, CCA, triangulation, evaluation of theory	Yes	26
Cohen, 2003	Accessing health services with hearing loss	Explore the subjective experiences of deaf and hard-of-hearing individuals undergoing psychotherapy	N=10 PHL students attending Gallaudet University	Semi-structured interviews	Not stated	No theoretical sampling no CCA, no simultaneous data collection & analysis or memoing	CCA, initial & focused coding	No	25
Cue et al, 2019	Identity and hearing loss: Meaning of hearing loss	Understand what it means to be D/deaf, and how it is influenced by developed identities	N=2 PHL (out of 15 participants, the rest identified as Deaf	Demographic questionnaire & narrative responses	Pragmatist school (implied)	No theoretical sampling, relationships between codes not explained in discussion, no theory evaluation	Reflexivity, theoretical saturation. CCA, initial, focused, and theoretical coding	Yes	33
Dillon & Pryce, 2019	Decision-making of hearing device usage	Identify patient preferences during decision making on whether to uptake or reject CI.	N=15 PHL with Post-lingual sensorineural hearing loss	Semi-structured interviews	Pragmatist school (implied)	No reflexivity, no memos, no evaluation of theory	Identified philosophical framework, theoretical sampling applied, theoretical saturation, CCA	Yes	29
Dorminy, 2014	Identity and hearing loss	Explore the deaf/ hard of hearing identity formation as non-signing PHL students integrate academically and socially	N=10 This study includes eight female and two male American students all with some form of	Structured interviews	Constructivist school (implied)	No theoretical sampling, no evaluation criteria applied	Memoing, member checking, CCA, initial coding, focused coding, simultaneous data collection	Yes	42

		into a predominantly signing Deaf university environment	hearing loss.				and analysis		
Dunsmore, Schneider, McKenzie & Gillespie (2020)	The impact of hearing loss on relationships and CPs	To develop a grounded theory of the social experiences of those with DSI, their family carer and their dyadic relationship	N=8 CPS 2 M: 6 F, age range 52-92. Mean = 73.4 years, SD 14.1 years	Intensive semi-structured interviews	Constructivist school	No reflection, or evaluation of theory	Analysis description, CCA, Memos, theoretical saturation	Yes	44
Fitzpatrick & Schramm, 2006	Healthcare professionals' perspectives on hearing loss considerations	Understand the experience of cochlear implantation in prelingual deafness from the perspective of service providers, i.e. audiologists	N=4 Four audiologists. The audiologists had a combined total of 21 years' experience in cochlear implants	Focus group & individual questionnaire Focus group & individual questionnaire	Stated constructivist school but followed are pragmatist school principles	No theoretical sampling, followed principles of school different to stated, no simultaneous data collection & analysis	Evaluation of theory, open, axial, and selective coding, CCA, and memoing	Yes	28
Fitzpatrick & Leblanc, 2010	Decision-making of hearing device usage	Investigate decision-making in cochlear implant patients- why they discontinue the use of hearing aids after implantation	N=12 PHL with post-lingual deafness	Semi-structured interviews & survey	Pragmatist school (implied)	No theoretical sampling, no axial or selective coding	Open coding, CCA, simultaneous data collection & analysis, member-checking, memoing	No	25
Gallagher & Woodside, 2018	Device adoption and usage	Identify factors affecting hearing aid adoption and use in adults with presbycusis	N=32 Three groups of older people with hearing loss in Northern Ireland were recruited:	Semi-structured interviews & demographic questionnaire	Constructivist school (implied)	No theoretical sampling, no simultaneous data collection & analysis, no memoing	Initial, focused, and theoretical coding, CCA	No	16

			(a) regular hearing aid users (n =12), (b) irregular hearing aid users (n = 10), and (c) hearing aid nonowners (n = 10).						
Gfeller, Driscoll, Schwalje, & 2019	Living with hearing loss: Experiencing music	Explore the perspectives of CI users on factors that support or undermine music experiences in everyday life.	N=40 PHL with cochlear implants	Semi-structured interviews & online questionnaire	Not stated	No theoretical sampling, no theoretical saturation, no theory evaluation	Reflexivity, philosophical framework, further literature review after theory development	Yes	33
Glade, 2017	The impact of hearing loss on relationships and CPs	Understand the role of spouses for recipients of CI users who were post-lingually deafened as well as the recipients of cochlear implants in auditory rehabilitation	N=12 Six couples, PHL with cochlear implant - severe to profound hearing loss & their married spouse.	Semi-structured interviews, intake questionnaire on impacts of hearing loss, observation notes, researcher's journal	Constructivist school (implied)	No theoretical sampling	Axial and selective codes, memoing, CCA, peer debriefing, persistent engagement (revisiting data for accuracy), reflection, audit trail, evaluation of theory, simultaneous data collection & analysis	Yes	36
Hallam, 2008	The impact of hearing loss on relationships and CPs	Explore the impact of acquired profound hearing loss (APHL) on the relationship between the PHL and their normally hearing close family	N=50 PHL with acquired profound hearing loss n = 25; communication partners: n=25	Semi-structured interviews	Pragmatist school (implied)	No theoretical sampling, no memos, no CCA, no simultaneous data collection & analysis, no evaluation of theory	Open, axial, and selective coding, CCA, reflection	Yes	26

		member, usually a partner, and identified the kinds of adjustment leading to maintenance or deterioration of the relationship							
Hallberg & Barrenas, 1993	The impact of hearing loss on relationships and CPs	Explore the perspective of spouses on their experiences of living close to a male with severe noise-induced hearing loss (NIHL)	N=10 All female CP-spouses of males with severe noise induced hearing loss (NIHL))	Thematised interviews	Pragmatist school (implied)	No theoretical sampling, no memos, no reflection	Open & selective coding, CCA, simultaneous data collection & analysis	Yes	25
Hallberg & Barrenas, 1995	Coping strategies for hearing loss	Identify the coping strategies in males for coping with noise induced hearing loss (NIHL)	N=53 PHL Men with NIHL	Semi-structured interviews	Glaserian school (implied)	No theoretical sampling, no reflection, no memos	Open, axial, and selective coding, CCA, some evaluation	Yes	17
Hallberg & Carlsson, 1991	Coping strategies for hearing loss	Identify the strategies individuals, develop for managing their hearing loss	N= 62 PHL Inductive Study- n=12 ; deductive study: =50	Semi-structured interviews	Glaserian school (implied)	No theoretical sampling, no memos, no reflection	Initial, focused coding, CCA	Yes	22
Hallberg & Jansson, 1996	The impact of hearing loss on relationships and CPs	Understand the perspective of women with NIHL, their experiences of noise as a threat to health and their having to live with a hearing disability	N=10 All females with NIHL	Semi-structured interviews	Glaserian school (implied)	No theoretical sampling, no memos, no reflection, no theory evaluation	Initial, focused coding, CCA, simultaneous data collection & analysis	Yes	24

Hallberg, Pike, & Ringdah, 2000	Coping strategies for hearing loss	Explore the perspective and coping abilities of individuals with pos-lingual severe-profound hearing impairment (>70dB LH at the frequency of 1kHz in the better ear), in everyday life	N=17 Post-lingual severe-profound hearing impairment. Hearing impairment since early childhood and all had hearing aids	Semi-structured interviews	Glaserian school (implied)	No reflection, no evaluation of theory	Theoretical sampling applied, data saturation, memoing, open, axial, and selective coding, CCA, simultaneous data collection, memos	Yes	30
Hallberg & Carlsson, 1993	Participation restrictions due to hearing loss	Identify the different situations that can lead PHL to feel disabled and experience participation restrictions in their daily lives.	N= 62 PHL Inductive Study-n=12 ; deductive study: =50	Semi-structured interviews	Pragmatist school (implied)	No theoretical sampling, no reflection, no memos	CCA, open coding, focused coding, cluster coding, theoretical coding.	Yes	21
Hallberg & Ringdahl, 2004	Living with hearing loss: Implications on everyday life	Investigate the implications of living with a cochlear implant on people with hearing loss in their day to day lives and quality of life	N=17 PHL with cochlear implants	Open interviews & field notes	Glaserian school (implied)	No evaluation of theory	ReflectioN , theoretical sampling applied, field notes, theoretical saturation, simultaneous data collection and analysis, interactional process, triangulation, memos, open, selective, theoretical coding, CCA	Yes	33
Hughes, 2001	Identity and hearing loss	Explore the self-concept of young PHL i.e. how they	N=9 PHL 1 mild to moderate, 1 moderate, 2 moderate	Semi-structured interviews	Glaserian school (implied)	Philosophical framework not reported	Theoretical sampling applied, reflection, theoretical sensitivity,	Yes	40

		perceive themselves	to severe, 2 severe, 3 severe to moderate. 8 Hearing aid users. 8 congenital HL, 1 late adolescence				memos, open, selective, theoretical coding, CCA, Evaluation of theory		
Hughes Hutchings, & Rapport, & 2018	Living with hearing loss: Social connectedness	Examine the perceptions of social connectedness and perceived listening effort in adult with cochlear implants	N=17 PHL: n=15 Post-lingual sensorineural hearing loss CP n=2: Significant others	Three focus groups & participant observation	Pragmatist school (implied)	No evaluation of theory	Theoretical sampling applied, memos, initial, focused, and theoretical coding, CCA, simultaneous data collection & analysis, reflexivity	Yes	36
Koerber, Moodie & Jennings (2021)	Occupational impacts of hearing loss	To investigate how nurses with hearing challenges adapt their telephone performance and workplace wellbeing in response to an online communication strategies training program.	N= 12 PHL nurses All female and over the age of 35 with eight over the age of 51	Semi-structured ethnographic interviews on telephone at baseline, and at a three-month follow up after course online communication training course.	Pragmatist school	No theoretical sampling, no reflection, no mention of theoretical saturation	Memos taken and Theory created	Yes	17

Laplante-Le´vesque, Pichora-Fuller, & Jean-Pierre Gagne, 2006	Audiological counselling and rehabilitation	Investigate the impact of an internet-based audiological counselling (i.e. emails sent from and to audiologist) on a new hearing aid user on their hearing rehabilitation journey	N=3 Adult new hearing aid users with bilateral hearing loss	Interviews & email exchange between audiologist and participant	Constructivist school (stated)	No theoretical sampling, no reflection, no memos	Open, selective & axial coding, CCA, member checking, triangulation	No	19
Lindqvist & Lundälv, 2012	Living with hearing loss: Work and transport	Explore the lived experiences of PHL's Participation in work Life and access to public transport	N=7 participants with a hearing disability	Three focus groups	Pragmatist school (implied)	No theoretical sampling, no reflection, no memos, no evaluation of theory, no theoretical saturation	Open & selective coding, CCA	Yes	16
Martin, 2010	Accommodations for PHL: Education facilities, communication support services	Investigate the perspectives of Albertan postsecondary students who are d/Deaf or hard of hearing and have accessed communication support services as part of their postsecondary experience	N=9 PHL with self-described HL: severe: n=2	Semi-structured interviews	Glaserian school (implied)	Different terminology for analysis than stated school (first, second level coding)	Theoretical sampling applied, reflection, memos, philosophical framework, CCA, evaluation of model, theoretical saturation	Yes	**47**
McRackan et al., 2017	Living with hearing loss: Quality of life	Develop a questionnaire that specifically measures the quality of life of cochlear implant users.	N=23 Cochlear implant users with bilateral severe to profound sensorineural hearing loss.	Three semi-structured focus groups	Constructivist school (stated)	No theoretical sampling, no reflection, no novel theory generated, no CCA, no in-depth coding, no discussion of theory or evaluation	Member checking, avoid bias, field notes	No	**9**

Ng, Phelan, Leonard & Galsterk, 2017	Device adoption and usage: Cochlear implant	Identify the uses and influences of using smartphone-connected hearing aids and its impact on patients, clinicians, and patient-clinician Interactions	N=19 Clinicians with experience using the connected hearing aid n=8 PHL n= 11	Semi-structured interview, purposive sampling of grey literature & triangulation	Not stated	No memos, no evaluation of theory	Theoretical sampling applied, reflection, initial, focused & theoretical coding, CCA, theoretical saturation, philosophical framework mentioned	Yes	35
Noonan, Gallor, Hensler-McGinnis, Fassinger, Wang, & Goodman, 2004	Living with hearing loss: Establishing successful careers	Investigate how highly achieving women with physical and sensory disabilities manage to achieve and create successful careers.	N= 1 PHL (one participant with hearing loss, amongst 17 participants with other disabilities)	Semi-structured interviews	Constructivist school (stated)	No memos	Reflexivity, field notes, initial & focused coding, theoretical saturation, evaluation of theory, CCA, triangulation, theoretical sampling	Yes	33
Pryce, Hall, Laplante-Levesque & Clark, 2016	Help-seeking behaviours for hearing loss	Examine decision making processes during help-seeking for adult hearing loss	N=27 PHL with presbycusis who do not require surgical management	Observation of patient/ audiologist encounters & focus group interviews	Constructivist school (stated)	No memos, no CCA, no evaluation of theory, no theoretical saturation	Theoretical saturation, initial and focused coding, reflexivity, theoretical sampling, simultaneous data collection & analysis, member checking, triangulation	Yes	25
Schoffstall et al., 2015	Healthcare professionals' perspectives on hearing loss	Identify how Vocational Rehabilitation Counselors (VRC) work with both d/Deaf and	N=10 Vocational Rehabilitation Counselors	Interviews	Pragmatist school (stated)	No theoretical sampling, no reflexivity, no CCA	Memos, open and selective coding, evaluation of theory	Yes	26

	considerations	hard of hearing consumers and their systemic environments to ensure that self-advocacy development takes place							
Shaw, Jennings, Poost-Forooshb, Hodginsa, & Kuchara, 2013	Healthcare professionals' perspectives on hearing loss consideration	Explore the need for networking and community to promote knowledge sharing and use between occupational therapists, audiologists, educators, regulators, workers, and employers regarding hearing loss	N=10 audiologists: n=5; occupational therapists: n=5	Semi-structured interviews	Pragmatist school (stated)	No reflexivity, no theoretical saturation	Evaluation of theory, CCA, memos, theoretical sampling, open & selective coding, member checking	Yes	30
Shaw, Tetlaffb, Jennings & Southalld, 2013	Living with hearing loss: occupational impacts	Investigate the impact of living with hearing loss on a PHL experience of the workplace	N=7 PHL with hearing loss between mild to profound	Semi-structured interviews	Pragmatist school (stated)	No theoretical sampling, no memos, no evaluation of theory	Reflexivity, theoretical saturation, open, axial & selective coding, CCA, simultaneous data collection, member checking	Yes	34
Shpigelman, & Gelkopf, 2017	Living with hearing loss: Safety during violence	Establish the impact of living with a disability, including hearing loss, on being exposed to politically violent events e.g. war	N=4 (out of 18 total sample with various	Three focus groups	Constructivist school (stated)	No theoretical sampling, no reflexivity, no memos, no theoretical saturation	Triangulation, member checking, axial coding, CCA	Yes	16

Svinndal, Jensen, & Rise, 2018	Living with hearing loss: occupational impacts	Investigate factors which facilitate or hinder participation at work, as described by PHL employees e.g. accommodation & leadership	N=21 PHL with long-term experience of hearing loss	Interviews	Pragmatist school (stated)	No evaluation of theory	Reflexivity, memos, theoretical sampling applied, theoretical saturation, simultaneous data collection and analysis, open & selective coding	Yes	34
Vieira, Dupas, & Chiari, 2018	Device adoption and usage: Cochlear implant	Identify the impact and effects of adult cochlear implantation on PHL	N=16 PHL with severe to profound hearing loss using cochlear implants	Semi-structured interviews	Pragmatist School (stated)	No reflexivity, no in-depth review of literature, no theory evaluation	Theoretical sampling applied, memos, theoretical saturation, CCA, conceptualization, categorization, and integration	No	28
Wallhagen, 2010	Living with hearing loss: Stigma	Examine the stigma of hearing loss from the perspectives of both people with hearing loss and communication partners	N=168 PHL= 91; CP=91 PHL who had limited HA usage or never worn them	Qualitative interviews	Not stated	No reflexivity, no theoretical saturation, no evaluation of theory, no initial sampling	Theoretical sampling, memos, member checking	Yes	14
**Total included in SR**		**39**				**SD: 8.5**			**Mean score: 26.5**
