# Supplementary material for: Applications of Grounded Theory Methodology to Investigate Hearing Loss: A Methodological Qualitative Systematic Review With Developed Guidelines
Source: Ear Hear. 2024 Apr 14;45(3):550–62. doi: 10.1097/AUD.0000000000001459 (PMC11008453; doi:10.1097/AUD.0000000000001459)
Supplement: Supplementary file 6 [file aud-45-550-s006.pdf]

Supplemental Digital Content 6: Guidelines showcasing 10 steps for grounded theory methodological excellence.

Guidelines for Grounded theory Applications for Researchers

1. *Choosing grounded theory*: Ensure that grounded theory is the most appropriate methodology to meet your research aims, and that a primary aim is to create a novel theory.
2. *Methodological self-consciousness*: engage in informed methodological decision making at every stage of your study.
3. *Philosophical Framework*: Outline the philosophical framework most appropriate for your research that will inform which grounded theory school to follow.
4. *Grounded theory school*: Choose which school to follow based on your research aims and overall philosophical stance.
5. *In-depth application of methodology:* Apply Grounded theory principles in all stages of study from design to analysis.
6. *Core grounded theory principles*: Ensure the application of all core grounded theory principles. These include theoretical sampling, constant comparative analysis, three-level theoretical coding, creating memos, simultaneous data collection & analysis, and reflexivity.
7. *Sample size*: Aim to recruit the recommended sample size for a grounded theory study, which is a minimum of 25 participants and continue recruitment until theoretical saturation is reached.
8. *Qualitative Rigour*: For developing a highly trustworthy and rigorous novel theory, engage in additional qualitative techniques that can be incorporated into grounded theory such as triangulation, member-checking, and crystallization.
9. *Evaluation of theory*: Use the relevant grounded theory evaluation criteria from your chosen school to evaluate your developed theory and establish its credibility and trustworthiness.
10. *Transparency in Publications*: Be transparent and explicit regarding the ways grounded theory was deployed across all stages in resultant publications for greater trustworthiness, even if mentions are brief due to word count limits. You can refer readers to supplementary files with more methodological information to overcome this.
